# GALV-KoRV-related retroviruses in diverse Australian and African rodent species

**DOI:** 10.1093/ve/veae061

**Published:** 2024-07-31

**Authors:** Joshua A Hayward, Shuoshuo Tian, Gilda Tachedjian

**Affiliations:** Life Sciences Discipline, Burnet Institute, 85 Commercial Rd, Melbourne, VIC 3004, Australia; Department of Microbiology, Monash University, Wellington Rd, Clayton, VIC 3168, Australia; Life Sciences Discipline, Burnet Institute, 85 Commercial Rd, Melbourne, VIC 3004, Australia; Life Sciences Discipline, Burnet Institute, 85 Commercial Rd, Melbourne, VIC 3004, Australia; Department of Microbiology, Monash University, Wellington Rd, Clayton, VIC 3168, Australia; Department of Microbiology and Immunology at the Peter Doherty Institute for Infection and Immunity, University of Melbourne, 792 Elizabeth St, Melbourne, VIC 3000, Australia

**Keywords:** cross-species virus transmission, Australian animal health, GALV gibbon-ape leukemia virus, KoRV koala retrovirus, phylogenetic analysis of endogenous retroviruses, NGS high-throughput sequencing data mining virus discovery

## Abstract

The enigmatic origins and transmission events of the gibbon ape leukemia virus (GALV) and its close relative the koala retrovirus (KoRV) have been a source of enduring debate. Bats and rodents are each proposed as major reservoirs of interspecies transmission, with ongoing efforts to identify additional animal hosts of GALV-KoRV-related retroviruses. In this study, we identified nine rodent species as novel hosts of GALV-KoRV-related retroviruses. Included among these hosts are two African rodents, revealing the first appearance of this clade beyond the Australian and Southeast Asian region. One of these African rodents, *Mastomys natalensis*, carries an endogenous GALV-KoRV-related retrovirus that is fully intact and potentially still infectious. Our findings support the hypothesis that rodents are the major carriers of GALV-KoRV-related retroviruses.

## Introduction

The origins of the gammaretroviruses gibbon ape leukemia virus (GALV) and koala retrovirus (KoRV) (referred to herein as GALV-KoRV-related retroviruses) have been a topic of sustained interest among the research community (Hanger et al. 2000, Hayward et al. 2013a, 2020, 2021, Simmons et al. 2014b, Xu and Eiden 2015, Denner 2016, Brown and Tarlinton 2017, McKee et al. 2017, Greenwood et al. 2018, McMichael et al. 2019, Mottaghinia et al. 2023). This is in part because KoRV and GALV (Hanger et al. 2000, Denner 2016), despite being closely related retroviruses, are hosted by koalas in Australia and have been reported in captive gibbons in Southeast Asia, respectively. The territories of koalas and gibbons do not overlap and are separated by a biogeographical faunal boundary across a large body of water, preventing direct viral transmission between these hosts (Brockelman and Geissmann 2020). In recent years, bats and rodents have become the prime suspects as transmitters of GALV-KoRV-related retroviruses across this region (Hayward et al. 2021, Mottaghinia et al. 2023), with multiple factors implicating these hosts. First, GALV-KoRV-related retroviruses were discovered in taxa other than koalas and gibbons in recent years, initially in an Australian rodent with subspecies in Indonesia (Simmons et al. 2014a, Alfano et al. 2016), and then in Australian and Asian bats whose geographic ranges link those of gibbons and koalas (McMichael et al. 2019, Hayward et al. 2020). Second, rodents and bats together comprise almost half of all mammalian species, and phylogenomic analyses have revealed that both bats and rodents are significantly involved in the history of retrovirus transmission between other mammalian species (Hayward et al. 2013a, Cui et al. 2015).

The phylogenetic tree of GALV-KoRV-related retroviruses remains fragmented, with apparent evolutionary gaps (Hayward et al. 2020). In particular, the evolutionary distance between KoRV, GALV, and other viral relatives is large enough that there are almost certainly other virus–host associations belonging to this clade that remain undiscovered. When considering the potential for zoonotic transmission and pathogenicity in humans or other animals of domestic, economic, and ecological importance, questions regarding the origin, transmission, and hosts of GALV-KoRV-related retroviruses need to be addressed.

Like many other retroviruses, KoRV and GALV are oncogenic, causing blood cancer in koalas and gibbons, respectively (Kawakami et al. 1980, Greenwood et al. 2018). KoRV is widespread in koala populations, particularly in northeastern Australia (Quigley et al. 2021b, Blyton et al. 2022a, 2022b, Vitali et al. 2023), and contributes to koala deaths through its association with diseases including chlamydia, the development of neoplastic lymphoma and immune modulation (Legione et al. 2017, Waugh et al. 2017, Maher et al. 2019, Sarker et al. 2020, McEwen et al. 2021, Imanishi 2023, McEwen and Greenwood 2023). GALV has been reported in captive gibbons, and the woolly monkey simian sarcoma virus (WMV SSAV, a strain of GALV referred to herein as WMV) was identified in a woolly monkey that had been housed with GALV-infected gibbons (Theilen et al. 1971, Wolfe et al. 1972).

Recently, a GALV-KoRV-related retrovirus was identified in a fruit bat with lymphoid leukemia (Van Brussel et al. 2023). Importantly, that virus, the Hervey pteropid gammaretrovirus (HPG), was previously shown to be capable of infecting human cells *in vitro* (Hayward et al. 2020). It remains an open question whether HPG or other GALV-KoRV-related retroviruses can establish an infection in humans.

Endogenous ‘fossil’ retrovirus sequences are ubiquitous within the genomes of mammals (Johnson 2019). This is a result of the hallmark of retrovirus replication where the retroviral proviral DNA precursor is inserted into the genome of the host (Johnson 2019). When this happens in germline cells that become new offspring, the retrovirus becomes an endogenized, heritable genetic element. Over the course of evolutionary history, vertebrate genomes have become littered with the remains of past retroviral infections (Johnson 2019). Endogenous retroviruses are subject to genetic drift and tend to become defective over many host generations (Johnson 2019). KoRV subtype A (KoRV-A) is a recently integrated endogenous retrovirus in the koala gene pool, and still generates infectious viral particles that can be transmitted between animals (Tarlinton et al. 2006, Quigley et al. 2021b). Other variants of KoRV (KoRV subtypes B-M) are understood to circulate among koalas as exogenous retroviruses (Joyce et al. 2021, Quigley et al. 2021a, Blyton et al. 2022a).

Here, we use the term ‘GALV-KoRV-related retroviruses’ to describe the endogenous and exogenous retroviruses which form a monophyly including GALV and KoRV, and which are not basal to or include the more distantly related *Mus caroli* endogenous retrovirus (McERV) (Hayward et al. 2020). Retroviruses within the GALV-KoRV-related retrovirus clade (with the noted exception of KoRV-B) use the PiT-1 receptor (SLC20A1) for cell entry (O’Hara et al. 1990, Xu et al. 2013, Denner 2016, Hayward et al. 2020). This receptor is ubiquitously expressed on mammalian cells, and a permissivity motif within its sequence can be used to infer potential susceptibility to infection by GALV-KoRV-related retroviruses (Schneiderman et al. 1996, Hayward et al. 2020). This clade includes, among others, the *Melomys burtoni* retrovirus (MbRV), Melomys woolly monkey retrovirus (MelWMV), as well as the newly reported complete Melomys woolly monkey retrovirus (cMWMV) hosted by Australian and New Guinean *Melomys* rodents, and HPG and the flying fox retrovirus (FFRV1) hosted by Australian bats (Simmons et al. 2014a, Alfano et al. 2016, Greenwood et al. 2018, McMichael et al. 2019, Hayward et al. 2020).

In this study, we aimed to leverage the vast amount of publicly available data in nucleotide sequence read archives (SRA) and genome assemblies to identify unreported GALV-KoRV-related retroviruses and their hosts. Novel retroviral sequences are phylogenetically analysed to infer their evolutionary relatedness to known gammaretroviruses, and the PiT-1 receptor sequences of their putative hosts are evaluated to infer their potential susceptibility to infection by GALV-KoRV-related retroviruses.

## Results

### GALV-KoRV-related retroviruses were identified in seven Australian rodent species

To identify GALV-KoRV-related retroviruses in all Australian mammals for which unassembled SRA have been made public, we searched for available SRA records for 80 species of Australian bats, 69 species of Australian rodents, 165 species of Australian monotremes and marsupials, and 8 species of other Australian eutherian mammals (SI Table 1). For many species, no publicly available SRA records exist. This was acute for Australian bats, with only 11 of 80 species (14%) represented. For other mammal groups, most species were represented among the SRA (SI Table 1): Rodents, 51/66 species (77%); Monotremes and marsupials, 130/165 species (79%); and other eutherian mammals, 8/8 (100%). BLAST hits were identified for one bat, one marsupial, and seven rodents (SI Table 1).

**Figure 1.**
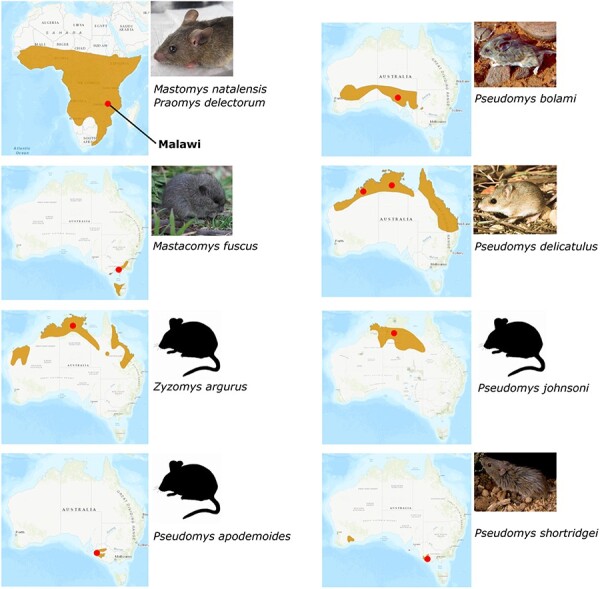
Rodent hosts of GALV-KoRV-related retroviruses. Australian and African rodents newly identified in this study as hosts of GALV-KoRV-related retroviruses are pictured. The natural ranges of these rodents are indicated by the brown-shaded region overlayed on the map of Africa or Australia (Aplin and Woinarski 2016, Burbidge and Woinarski 2016, Cassola 2016, Cassola and Menkhorst 2016, Granjon 2016, Burbidge 2016a, 2016b, Woinarski and Burbidge 2016a, 2016b). The approximate locations of the collected samples are indicated by the dots.

The initial search query in our iterative search strategy was a 540-nt sequence from the receptor binding domain of a GALV-KoRV-related retrovirus (SI Data 2). Consistent with previous findings, BLAST hits from the bat *Pteropus poliocephalus* (Grey-headed flying fox) matched the known GALV-KoRV-related retrovirus HPG (Hayward et al. 2020, Van Brussel et al. 2023), and the marsupial *Phascolarctos cinereus* (koala) was positive for KoRV. Hits representing novel retroviral sequences were from the rodents *Mastacomys fuscus* (Broad-toothed rat)*, Pseudomys apodemoides* (Silky mouse)*, P. bolami* (Bolam’s mouse)*, P. delicatulus* (Delicate mouse)*, P. johnsoni* (Central pebble-mound mouse)*, P. shortridgei* (Heath mouse), and *Zyzomys argurus* (Common rock rat). These rodents occupy diverse regions across the Australian continent that collectively include all Australian states and the Northern Territory (Fig. 1). SRA reads from each species were extracted and *de novo* assembled into contigs (∼200–1000 nt) for subsequent alignment and phylogenetic analyses (Table 1A). Some contigs from individual species were found to overlap. Overlapping contigs likely represent either different retroviruses or multiple germline insertions/duplications of the same retrovirus whose sequences have diverged over time (Johnson 2019).

‘*Mastomys natalensis*, aka the natal multimammate mouse’ by JD Kelly et al. (Kelly et al. 2013), available at https://upload.wikimedia.org/wikipedia/commons/1/18/Mastomys_natalensis.jpg under a Creative Commons Attribution 3.0; ‘Broad-toothed mouse (*Mastacomys fuscus*); Thredbo, NSW, Australia’ by M Kjaergaard, available under a Creative Commons Attribution 3.0 at https://upload.wikimedia.org/wikipedia/commons/2/24/Broad_toothed_mouse_1.JPG; ‘Bolam’s mouse (*Pseudomys bolami*)’ by A Stanley, available under a Creative Commons Attribution 4.0 at https://upload.wikimedia.org/wikipedia/commons/4/46/Bolam%27s_mouse_%28Pseudomys_bolami%29_.jpeg; ‘Little Native Mouse’, by ZooPro, available under a Creative Commons Attribution 3.0 at https://upload.wikimedia.org/wikipedia/commons/0/04/Pseudomys_delicatus_20020401.jpg; ‘Heath mouse, native Australian rodent at Black Range State Park’ by David Paul, available at https://upload.wikimedia.org/wikipedia/commons/8/81/Pseudomys_shortridgei%2C_Heath_mouse_01.jpg under a Creative Commons Attribution 4.0.

**Table 1.** Mammalian species samples in which novel GALV-KoRV-related retroviruses were identified using the Hervey pteropid gammaretrovirus receptor binding domain nucleotide sequence.

	Species	Common name	Range	^a^ Hits to HPG RBD Genbank SRA and WGS accessions	ORF(s) intact?	Bioproject Genbank accession	^b^ Most similar GKRR	^h^ Specimen source	Source citation
**A**	*Mastacomys fuscus*	Broad-toothed rat	NSW, VIC, TAS	SRX8933290^c^	N	PRJNA512907	WMV	MVC, Victoria, 2013	n/a
				SRX10229751	N	PRJNA705792	WMV	ABTC, Kosciusko National Park, NSW	Roycroft et al., 2020
	*Pseudomys apodemoides*	Silky mouse	SA, VIC	SRX10921805^d^	N	PRJNA729818	GALV	ABTC, South Australia	Roycroft et al., 2021
	*Pseudomys bolami*	Bolam’s mouse	SA, WA, NSW	SRX11072699^e^	N	PRJNA729818	WMV	SAMC, South Australia	Roycroft et al., 2021
	*Pseudomys delicatulus*	Delicate mouse	WA, NT, QLD	SRX10921811^f^	Y	PRJNA729818	WMV	ABTC, Northern Territory	Roycroft et al., 2021
				SRX11072702	Y	PRJNA729818	WMV	WAMC, Western Australia	Roycroft et al., 2021
	*Pseudomys johnsoni*	Central pebble-mound mouse	WA, NT, QLD	SRX10921824	N	PRJNA729818	WMV	ABTC, Northern Territory	Roycroft et al., 2021
	*Pseudomys shortridgei*	Heath mouse	WA, SA, VIC	SRX11072725^g^	N	PRJNA729818	WMV	ABTC, Victoria	Roycroft et al., 2021
	*Zyzomys argurus*	Common rock rat	WA, NT, QLD	SRX10921833	N	PRJNA729818	WMV	ABTC, Northern Territory	Roycroft et al., 2021
**B**	*Mastomys natalensis*	Natal multimammate mouse	Sub-saharan Africa	JADRCE01021795	Y	PRJNA669840	WMV	Malawi, 2007	Kumon et al., 2021
	*Praomys delectorum*	Delectable soft-furred mouse	East Africa	JADRCD010013576	N	PRJNA669840	WMV	Malawi, 2007	Kumon et al., 2021

a The Hervey pteropid gammaretrovirus (HPG) receptor binding domain (RBD) was used to identify matching reads or contigs in the listed sequence read archives (SRA) or whole-genome shotgun (WGS) contig archives.

b The most similar GALV-KoRV-related retrovirus (GKRR) to the identified reads/contigs was determined by sequence alignment. The most similar GKRR was used as a search query to identify additional reads in other SRA.

c G Additional reads were identified for C Mastacomys fuscus, SRX11072735, SRX10921840, SRX10921839.

d Pseudomys apodemoides, SRX11072696.

e Pseudomys bolami, SRX11072698, SRX10921807.

f Pseudomys delicatulus, SRX11072703, SRX11057250, SRX10921812 .

g Pseudomys shortridgei, SRX11072727, SRX10921831, SRX10921832.

h If no specimen source collection date is listed, then it is unknown.

ABTC, Australian Biological Tissues Collection; GALV, gibbon-ape leukemia virus; KoRV, koala retrovirus; MVC, Museums Victoria Collection; NSW, New South Wales; NT, Northern Territory; ORF, open-reading frame; QLD, Queensland; SA, South Australia; SAMC, South Australia Museum Collection; Tas, Tasmania; VIC, Victoria; WA, Western Australia; WAMC, Western Australia Museum Collection; WMV, wooly monkey virus.

**Figure 2.**
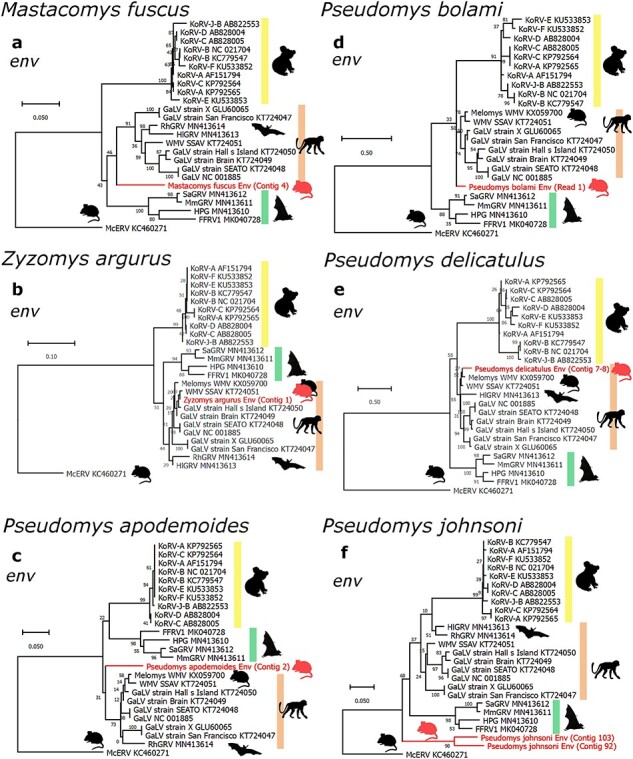
Phylogenetic evolutionary analysis of Australian rodent GALV-KoRV-related retroviral sequences. Maximum likelihood phylogenies of regions of nucleotide sequences from the *env* genes of (a) *Mastacomys fuscus*; (b) *Zyzomys argurus*; (c) *Pseudomys apodemoides*; (d) *Pseudomys bolami*; (e) *Pseudomys delicatulus*; and (f) *Pseudomys johnsoni*. Shading indicates sub-clades within the GALV-KoRV-related retrovirus clade. The KoRV, GALV/WMV, and HPG sub-clades are shaded yellow, orange, and green, respectively. Silhouettes represent the animal hosts rodents, koalas, primates, fruit bats, and microbats. All branches are scaled according to the number of nucleotide substitutions per site as indicated by the scale bars. Trees were rooted using the McERV (*Mus caroli* endogenous retrovirus) KC460271. Bootstrap support values are shown at the nodes. The number of nucleotide positions in the multiple sequence alignments used to generate phylogenies (a–f) are 523, 175, 172, 101, 476, and 150 respectively.

Contigs whose most similar match was a GALV-KoRV-related retrovirus, as determined by BLAST, were phylogenetically analysed (Fig. 2, SI Figs 1–7). The nucleotide percentage identities for each contig compared to GALV, WMV, and KoRV-A are provided in SI Table 2. Overlapping contigs from individual species were included in the same phylogenies, while contigs that did not overlap were analysed separately. Phylogenies for *gag, pol*, and *env* retroviral sequences from each rodent species are included in SI Figs1–4. Additional phylogenies for several of these species are included in SI Fig 5–7. Most contigs assembled from rodent SRA contained deleterious mutations such as frameshifting indels or premature stop codons (Table 1A, SI Data 1). This indicates that these contigs were derived from defective endogenous retroviruses. Only one species, *P. delicatulus*, yielded contigs that did not contain deleterious mutations (Table 1A).

All Australian rodent datasets contain multiple contigs from one or more of the core retroviral genes (*gag, pol*, and *env*) that phylogenetically clustered within the GALV-KoRV-related retrovirus clade. (Fig. 2, SI Figs 1–7). No contigs were identified which were closely and immediately basal to the KoRV sub-clade. For *env* sequences (Fig. 2), contigs clustering within the GALV/WMV sub-clade were derived from *Z. argurus* and *P. delicatulus* (Fig. 2b and e). *Env* contigs adjacent to the GALV/WMV sub-clade were derived from *M. fuscus, P. bolami, P. apodemoides* (Fig. 2a, c, and d), and *P. shortridgei* (SI Fig. 4C). *Env* contigs basal to all other GALV-KoRV-related retroviruses were derived from *P. johnsoni* (Fig. 2f).

For *gag* and *pol*, contigs clustered at variable positions within the phylogeny, including in some cases, at a position basal to McERV (SI Figs 1–7). Together, these contigs may represent multiple divergent gammaretroviruses, or individual recombinant retroviruses that include one or two genes derived from a GALV-KoRV-related retrovirus, and the remainder from a more distantly related gammaretrovirus. Taken together, these data implicate three novel genera of Australian rodents (*Pseudomys, Mastacomys*, and *Zyzomys*) as hosts of GALV-KoRV-related retroviruses, in addition to the *Melomys* genus.

### African rodents harbour GALV-KoRV-related retroviruses, one of which is intact and potentially infectious

We extended our search for unreported GALV-KoRV-related retroviruses to all parts of the world, searching within all mammalian species for which genome assemblies have been made public. BLAST hits were identified for numerous taxa within the Laurasiatheria (e.g. bats) and Eurachontoglires (e.g. rodents) superorders, while no hits were identified within primates, Afrotheria, Xenarthra, Marsupialia (except koalas), or Monotremata (SI Table S3). The RefSeq/WGS contig hits were extracted and annotated for subsequent analyses (Table 2).

**Table 2.** Mammalian ERVs identified in WGS assemblies using the Hervey pteropid gammaretrovirus receptor binding domain nucleotide sequence.

Family	Species	Common name	Range	WGS Contig Genbank Accession	ERV coverage (nt)	^a^ Complete ERV present	^b^ ERV Intact	5ʹLTR	*gag*	*pol*	*env*	3ʹLTR
Carnivora	*Mustela putorius furo*	Ferret	Europe	JAADYL010000761	9075	Y	N	F	D	D	D	F
	*Spilogale gracilis*	Western spotted skunk	N America	PITA01038320	7878	N	N	P	D	D	D	P
	*Spilogale interrupta*	Plains spotted skunk	N America	JAKZGT010000455	8680	Y	N	F	D	D	D	F
Chiroptera	*Megaderma lyra*	Greater false vampire bat	South & SE Asia	AWHB01177640	1480	N	N	B	B	P, I	P, D	B
				AWHB01450121	1097	N	N	B	B	P, I	P, D	B
	*Murina aurata feae*	Fea’s tube-nosed bat	East & SE Asia	PVJC01052402	8448	Y	N	F	D	D	D	F
				PVJC01075163	4875	N	N	B	P, D	D	D	F
				PVJC01084952	4433	N	N	B	B	D	P, D	B
Dermoptera	*Galeopterus variegatus*	Sunda flying lemur	SE Asia	PVJT020008927	3049	N	N	B	B	P, D	D	F
Eulipotyphla	*Scalopus aquaticus*	Eastern mole	N America	PVIJ01008085	6806	N	N	B	B	P, D	D	F
	*Suncus etruscus*	Etruscan shrew	N Africa, Eurasia & SE Asia	JAMYDY010013653	5383	Y	N	F	D	D	D	F
Lagomorpha	*Lepus timidus*	Mountain Hare	Eurasia	VUAX01000454	10 574	Y	N	F	D	D	D	F
	*Oryctolagus cuniculus*	European rabbit	Europe & Australia	AAGW02069446	8621	Y	N	F	I	D	I	F
Pholidota	*Manis javanica*	Sunda pangolin	SE Asia	JAMQTK010001946	8685	Y	N	F	D	D	D	F
Rodentia	*Apodemus speciosus*	Large Japanese field mouse	Japan	BDUI01000676	9323	Y	N	F	D	D	D	F
				BDUI01030468.1	5856	Y	N	F	D	D	D	F
	*Apodemus sylvaticus*	Wood mouse	Europe	LIPJ01004397	3497	N	N	B	B	P, D	D	P
				LIPJ01009012.1	3856	N	N	B	B	P, D	D	P
				LIPJ01031882	8778	Y	N	F	D	D	D	F
	*Arvicanthis niloticus*	African grass rat	Africa	JAAOMF010000019.1	6160	Y	N	F	D	D	D	F
				JAAOMG010000022.1	6187	Y	N	F	D	D	D	F
	*Mastomys coucha*	Southern multimammate mouse	S Africa	VSBT01000014.1	2646	N	N	B	B	P, D	D	F
	*Mastomys natalensis*	Natal multimammate mouse	Sub-saharan Africa	JADRCE010217952	8461	Y	Y	F	I	I	I	F
	*Praomys delectorum*	Delectable soft-furred mouse	E Africa	JADRCD010013576	1550	N	N	B	B	P, I	D	B
	*Sigmodon hispidus*	Hispid cotton rat	N America	PVIH01025626	8167	N	N	B	D	D	D	F
				PVIH01054863	1665	N	?	B	B	P, I	P, I	B
Scandentia	*Tupaia tana*	Large treeshrew	SE Asia	RJWV010071866	6753	N	N	B	B	D	D	F

a Whole genome shotgun (WGS) assembly contigs are variable in size. Many are shorter than the endogenous retroviruses (ERVs) present within them. Whether or not the contig contains the complete ERV is indicated.

b Whether or not the ERV is intact is indicated. Intact means that all regions are present and there are no obviously deleterious mutations such as frameshift indels or premature stop codons. The status of each region within the ERV is indicated as follows: B (Beyond), Entire region is beyond end of the contig; D (Defective), Region is fully contained within the contig and contains deleterious mutations; F, (Full) Region is fully contained within the contig; I (Intact), Region is fully contained within the contig and there are no obviously deleterious mutations; P (Partial), Region is partially present and the remainder is beyond end of contig. LTR, long terminal repeat.

**Figure 3.**
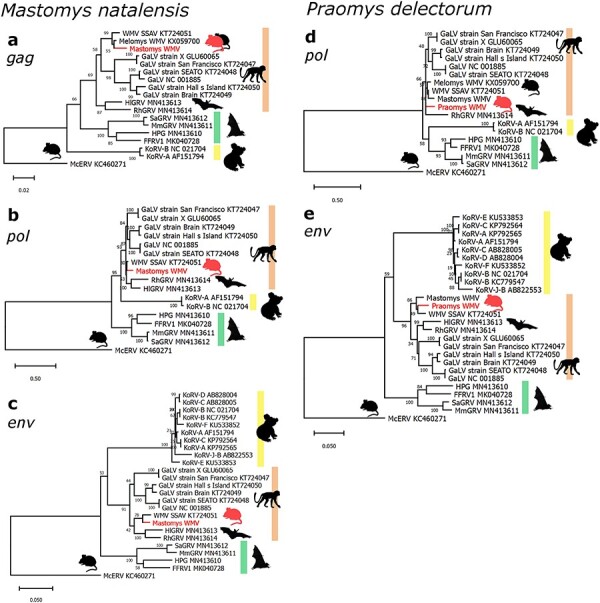
Phylogenetic evolutionary analysis of African *Mastomys natalensis and Praomys delectorum* GALV-KoRV-related retroviruses. Maximum likelihood phylogenies of the (a), *gag*, (b,d) *pol*, and (c,e) *env* genes of GALV-KoRV-related retroviruses and the novel (a–c) Mastomys WMV (*Mastomys* woolly monkey virus) and (d–e) Praomys WMV. Shading indicates sub-clades within the GALV-KoRV-related retrovirus clade. The KoRV, GALV/WMV, and HPG sub-clades are shaded yellow, orange, and green, respectively. Silhouettes represent the animal hosts rodents, koalas, primates, fruit bats, and microbats. All branches are scaled according to the number of nucleotide substitutions per site as indicated by the scale bars. Trees were rooted using the McERV (*Mus caroli* endogenous retrovirus) KC460271 sequences. Bootstrap support values are shown at the nodes. The number of nucleotide positions in the multiple sequence alignments used to generate phylogenies (a–e) are 852, 1904, 1049, 446, and 1119, respectively.

The sequences identified in genome assemblies represent more complete (1097–10 574 nt) endogenous retroviruses as they were extracted from larger genome assembly contigs compared to the short contigs assembled for the Australian rodents from SRA. Among the endogenous retroviruses identified in this search, two were found to phylogenetically cluster within the GALV-KoRV-related retrovirus clade (Fig. 3, Tables 1B and 2). These GALV-KoRV-related retroviruses are hosted by the African rodents *Mastomys natalensis* and *Praomys delectorum*.

**Figure 4.**
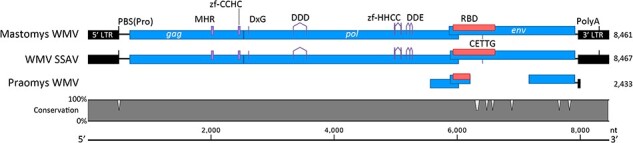
Alignment of the Mastomys WMV, Praomys WMV, & WMV SSAV. Open reading frames encoding the core retroviral genes *gag, pol*, and *env*, and the 5ʹ and 3ʹ long terminal repeats (LTR) are depicted. The alignment scale is in nucleotides and the total length of each sequence is listed on the right side of the alignment. The Conservation line graph (grey region) depicts nucleotide mismatches between Mastomys WMV and WMV SSAV. Conserved functional motifs (purple regions) are indicated: PBS(Pro), proline tRNA primer-binding site; MHR, major homology region; zf, zinc finger; DxG, protease active site motif; DDD, reverse transcriptase active site motif; DDE, integrase active site motif; RBD, receptor binding domain; CETTG, pathogenicity motif; PolyA, polyadenylation signal; *env*, envelope; *gag*, group-specific antigen; Mastomys WMV, *Mastomys* woolly monkey virus; WMV SSAV, woolly monkey simian sarcoma virus; *pol*, polymerase.

The first African GALV-KoRV-related retrovirus, derived from *M. natalensis*, is completely intact (Fig. 4) and has a high 96.9% nucleotide sequence identity to WMV. We have designated this ERV as Mastomys WMV according to the convention established by Melomys WMV (Alfano et al. 2016), and alternatively as ERV-WMV.1-Mna according to the broader convention for ERV designation (Gifford et al. 2018).

For comparison, Mastomys WMV shares 79.9% and 88.7% nucleotide identity with KoRV-A and GALV, respectively. Whole genome nucleotide phylogenetic analysis of complete and near complete gammaretroviruses places Mastomys WMV immediately basal to WMV, Melomys WMV, and cMWMV (SI Figure 8A). Phylogenetic estimation of the relationship of Mastomys WMV to MbRV, which is comprised of short fragments of *pol* and *env*, was performed separately to include MbRV, and likewise indicates that Mastomys WMV is basal to other WMV variants (SI Figure 8B). Mastomys WMV contains the expected open reading frames for *gag, pol*, and *env* and all the expected functional motifs are conserved (Fig. 4). These include: the proline primer binding site and polyadenylation signal, the major homology region and CCHC zinc finger of Gag, and the protease, reverse transcriptase, and integrase enzymatic active sites (Fig. 4). In addition, many of the nucleotide differences in the receptor binding domain compared to WMV are non-synonymous mutations, and the CETTG pathogenicity motif is present in *env* (Fig. 4 & SI Figure 9).

The 5ʹ and 3ʹ long terminal repeats (LTRs) of Mastomys WMV are 100% identical (Fig. 4). The lack of nucleotide differences resulting from genetic drift since integration indicates that this retrovirus integrated into the genome of *M. natalensis* recently. The presence of intact *gag, pol*, and *env* genes and functional enzymatic motifs suggests that Mastomys WMV can potentially express infectious viral particles.

Two genome assemblies are available for *M. natalensis*: ASM1984379v1 (Genbank GCA_019843795.1) and UFL_Mnatal_1.0 (Genbank: GCA_021653895.1). Mastomys WMV is present as a single ERV in the ASM1984379v1 assembly, derived from a specimen collected in Malawi in 2007, but is absent from UFL_Mnatal_1.0, derived from a specimen collected in Eswatini in 2019. This indicates that Mastomys WMV is unfixed in the *M. natalensis* gene pool.

The second African GALV-KoRV-related retrovirus, derived from *P. delectorum*, and designated as Praomys WMV/ERV-WMV.1-Pde, was identified on a relatively short genomic contig comprising the 3ʹ end of the *pol* gene, and the 5ʹ and 3ʹ ends of the *env* gene with an 889 nt internal deletion relative to WMV (Fig. 4). Aside from this deletion, Praomys WMV contains mostly uninterrupted reading frames. Taken together, this indicates that this is a defective endogenous retrovirus. It shares 95.2% nucleotide identity with WMV and is phylogenetically basal to previously reported WMV variants (Fig. 3, SI Figure 8B).

### Novel hosts of GALV-KoRV-related retroviruses possess the PiT-1 cell receptor permissivity motif

We analysed the PiT-1 sequences for all the novel hosts of GALV-KoRV-related retroviruses reported here to determine if they possessed any mutations that might indicate evolutionary adaptation towards non-permissivity. All but one rodent PiT-1 sequence from *Z. argurus*, which contains a codon deletion, possesses the permissive motif (SI Figure 10). This suggests that these rodents are likely susceptible to cell entry by GALV-KoRV-related retroviruses engaging the PiT-1 receptor, with the possible exception of *Z. argurus* (SI Figure 10).

### Phylogenetic analysis of clade-adjacent endogenous retroviruses suggests a potential rodent origin for GALV-KoRV-related retroviruses

**Figure 5.**
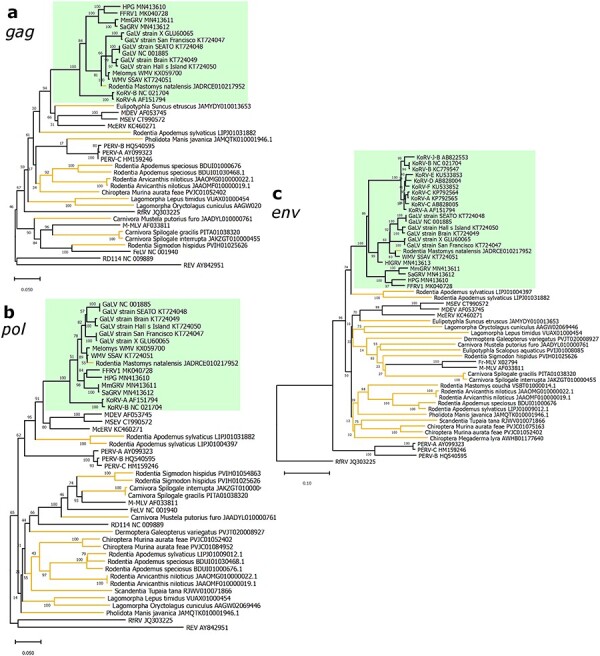
Evolutionary relationships of novel endogenous gammaretroviruses identified in mammalian genome assemblies via sequence homology with a GALV-KoRV-related retroviral receptor binding domain sequence. Maximum likelihood phylogenies of the (a) *gag*, (b) *pol*, and (c) *env* genes. All branches are scaled according to the number of nucleotide substitutions per site as indicated by the scale bars. The GALV-KoRV-related retrovirus clade is shaded in green; branches representing novel endogenous retroviruses reported in this study are shown in orange; branches representing previously reported gammaretroviruses are shown in black. Bootstrap support values are shown at the nodes. The number of nucleotide positions in the multiple sequence alignments used to generate phylogenies (a–c) are 731, 700, and 1260, respectively.

Phylogenetic analysis of the endogenous retroviruses extracted from RefSeq/WGS genome assemblies revealed that most of the endogenous retroviruses identified through sequence homology with the GALV-KoRV-related receptor binding domain were gammaretroviruses outside the GALV-KoRV-related retrovirus clade (Fig. 5). This analysis also suggested a geographic bias among the hosts of these endogenous retroviruses (Fig. 5 & Table 2). Using our targeted search strategy, 27 endogenous retroviruses were identified across 19 species (Table 2). Of these, six species are present in Southeast Asia, and one in Australia, comprising 37% of the host species. These Southeast Asian host species are diverse and include microbats (*Megaderma lyra* and *Murina aurata feae*), lemurs (*Galeopterus variegatus*), Etruscan shrews (*Suncus etruscus*), pangolins (*Manis javanica*), and treeshrews (*Tupaia tana*). Further, among the identified host taxa, 7 of the 19 (37%) species are rodents (Table 2). These data indicate that despite not phylogenetically clustering within the GALV-KoRV-related retrovirus clade, a large proportion of gammaretroviruses with receptor binding domain sequence homology to GALV-KoRV-related retroviruses are hosted by Southeast Asian mammals and the host species are predominantly rodents.

## Discussion

KoRV and its close relatives are viruses of ecological concern, with origins shrouded in mystery, and potentially pathogenic consequences in the event of further cross-species transmission events into humans or other animals. To enhance our understanding of the breadth of the host network of this group of viruses and identify hosts which may require further attention, we searched for previously unreported GALV-KoRV-related retrovirus sequences hidden in the growing expanse of publicly available sequence data.

### GALV-KoRV-related retroviruses in multiple species of Australian rodents

We identified GALV-KoRV-related retroviral sequences in seven species of Australian rodents (Table 1A). All SRA records in which these retroviral sequences were identified were exomes recently generated from Australian museum specimens (Roycroft et al. 2020, 2021). These rodent species inhabit diverse territories around much of coastal Australia, including Tasmania (Fig. 1). KoRV itself is present in koalas from Queensland, New South Wales, and to a lesser extent in Victoria and South Australia (Blyton et al. 2022a). However, reported rodent GALV-KoRV-related retroviruses in Australia had previously been limited to the host *Melomys burtoni*, which ranges in the north and northeastern coast of Australia (Aplin et al. 2016). This geographic distribution suggests that GALV-KoRV-related retroviruses are widespread across Australia and may infect many more hosts than previously known.

Given the limited read depth of the rodent SRA datasets, only short contigs could be assembled from reads with sequence homology to GALV-KoRV-related retroviruses. Despite this limitation, deleterious mutations in these retroviruses were sufficiently abundant that almost all contigs were found to contain them. This suggests that they are likely endogenous retroviruses and that their integration was not recent. Complete endogenous retroviral genomes from these rodents will help to determine how long ago these integration events occurred.

Many of the assembled contigs overlapped in their positions of alignment to GALV-KoRV-related retroviral genomes. In these cases, they could be included in the same phylogenies (Fig. 2f, SI Figs 1–3, 5–7). Overlapping but distinct contigs indicate the presence of either multiple retroviruses or duplication of a single integrated retrovirus followed by sequence divergence (Johnson 2019). Clarifying this will require more extensive sequencing of these rodent genomes. It is also worth noting that five of the seven Australian rodent species are members of the same genus, *Pseudomys*. Together with the observation that these retroviral sequences are likely not recent integrations, this might suggest that one or more of the endogenous retroviruses represented by these contigs integrated into a common ancestor of these rodents.

For each Australian rodent species, the GALV-KoRV-related retroviral contigs, representing the *gag, pol*, and *env* genes, appeared at varying positions in the phylogenies relative to the KoRV, GALV/WMV, and HPG sub-clades (Fig. 2, SI Figs 1–7). This may indicate that the analysed *gag, pol*, and *env* contigs are derived from different endogenous retroviruses, or that the limited sequence information in these relatively short contigs prevents robust estimation of evolutionary relationships. The latter possibility is suggested by the weak bootstrap support of some internal nodes across the phylogenies (Fig. 2, SI Figs 1–7). Another important possibility is that some of the contigs may represent ERVs that have arisen through the recombination of different retroviruses. Retroviral recombination is a well understood mechanism of retroviral diversification (Negroni and Buc 2001). For example, a sub-clade of Type D betaretroviruses which includes primate and bat retroviruses arose through a recombination event that joined a gammaretroviral env region to a betaretroviral gag-pol region (Hayward et al. 2013b).

### Identification of GALV-KoRV-related retroviruses in African rodents

Surprisingly, we identified GALV-KoRV-related retroviruses in the genomes of two African rodents. To our knowledge, this is the first report of GALV-KoRV-related retroviruses infecting hosts outside of Australia and Southeast Asia. Both endogenous retroviruses appear to be very recently integrated into the *M. natalensis* and *P. delectorum* genomes. This is evidenced by the 100% nucleotide identity between the Mastomys WMV long-terminal repeat regions and a very low frequency of indel mutations in Praomys WMV.

Mastomys WMV has a fully intact proviral genome. Previously reported GALV-KoRV-related retroviruses from rodents have contained incomplete or otherwise defective genomes. The only exception to this is cMWMV, which is a newly reported, infectious, non-fixed endogenous GALV-KoRV-related retrovirus present in a subset of individuals of the New Guinea rodent species *Melomys leucogaster* (Mottaghinia et al. 2024). All canonical genes are present in Mastomys WMV, with uninterrupted open reading frames and the conservation of expected functional motifs (Fig. 4). The presence of multiple non-synonymous mutations in the receptor binding domain is consistent with ongoing selective pressures in different hosts following the divergence of WMV and Mastomys WMV from their common ancestor. Mastomys WMV is present in one of two available genome assemblies for *M. natalensis*. These assemblies are derived from specimens collected in different locations on the African continent, Malawi and Eswatini, ∼2000 km apart. This indicates that Mastomys WMV is not fixed in the *M. natalensis* gene pool, suggesting that it may be actively in the process of endogenization and fixation, similar to KoRV. If rodents are the major hosts of this clade of retroviruses and responsible for many interspecies transmission events, then the identification of potentially infectious rodent GALV-KoRV-related retroviruses fills an important gap in supporting this notion.

Mastomys WMV may reflect a similar case to KoRV-A and cMWMV in which the virus, being recently integrated, is in the process of endogenization and fixation in the gene pool while still producing functional, infectious retroviral particles. Extended sampling and analysis of Mastomys WMV presence among individual *M. natalensis* rodents may clarify this possibility. Praomys WMV may also be in the process of undergoing endogenization and fixation in its murine host; however, the identified copy is clearly no longer functional since it has a deletion of 889 nt (relative to WMV) in the *env* gene that would prohibit this endogenous retrovirus from generating infectious viral particles. Mastomys WMV and Praomys WMV appear to be distinct retroviruses, as each has higher nucleotide identity to WMV than to each other (94.3%), suggesting that they are not the result of a single integration event prior to speciation of the *Mastomys* and *Praomys* hosts.

The high degree of nucleotide identity of these African retroviruses to WMV suggests recent transmission. Rodents of various species have been documented as pests aboard ships (Song et al. 2003, Harding et al. 2023). There are shipping routes across the Indian Ocean connecting Southeast Asia, Australia, and East Africa, and it seems reasonable to speculate that a rodent stowaway carrying an infectious WMV variant might explain these findings. The newly reported cMWMV shares exceptionally high nucleotide identity with WMV (98.9%) (Mottaghinia et al. 2024), which is closer than the nucleotide identity shared between Mastomys WMV, Praomys WMV, and WMV. Taken together, these studies suggest that the WMV sub-clade is actively undergoing transmission between rodent species/genera and geographic regions.

### Possible rodent origins of GALV-KoRV-related retroviruses

We searched publicly available genome assemblies for evidence of endogenous retroviruses belonging to the GALV-KoRV-related retrovirus clade using a stringent search query comprised of the receptor binding domain of a GALV-KoRV-related retrovirus. This did not fully exclude retroviruses from outside this clade from appearing among the BLAST hits. Perhaps unsurprisingly, a substantial proportion (37%) of the hosts of the identified retroviruses are found across Southeast Asia and Australia (Table 2). Similarly, many (37%) of these hosts are rodents (Table 2).

While the *gag* and *pol* genes of these endogenous retroviruses are widely dispersed throughout the gammaretroviral phylogeny (Fig. 5a and b), the *env* genes cluster within a sister clade to the GALV-KoRV-related retroviruses (Fig. 5c). This suggests a history of recombination between GALV-KoRV-related retroviruses and more distantly related gammaretroviruses. Furthermore, all extant retroviruses within the *env* sister clade are rodent gammaretroviruses such as the Moloney murine leukemia virus (MMLV) and *Mus caroli* endogenous retrovirus (McERV). These are hosted by Asian mice, some of which are now globally distributed.

It is worth noting that although GALV/WMV is a prominent, defining group within the GALV-KoRV-related retrovirus clade, no new GALV-KoRV-related retrovirus sequences were identified in any novel primate hosts. Further, no GALV-KoRV-related ERVs have been reported in any primate host. Based on the currently available data, this suggests that primates are an incidental host to which GALV/WMV has been recently transmitted and are not the original or natural hosts of this clade of retroviruses.

An important caveat here is the possibility of sampling bias among the genomes represented in the public datasets we queried influencing the apparent abundance of GALV-KoRV-related retroviruses in Southeast Asian species relative to other locations. Mammalian species from some geographical regions are likely to be better represented among genome datasets than others. It is worth noting that at present, the RefSeq database (Release 212) contains 1443 mammalian genomes, which represents only ∼22% of the currently recognized 6495 mammalian species (Burgin et al. 2018, NCBI 2022). Future analyses that include a larger, more comprehensive, and geographically unbiased collection of reference mammalian genomes may clarify this possibility. Intriguingly, the new report by Mottaghinia et al. (Mottaghinia et al. 2024) suggests an Australo-Papuan origin for this clade (therein referred to as ‘GALV-like’ retroviruses) following the identification of vertically transmitted ERVs in *Melomys* rodents which, as with KoRV, is at the earliest stages of endogenization. Here we report an additional recent endogenization in the African rodent *M. natalensis*. The picture of modern GALV-KoRV-related retroviral infection and endogenization is clearly far from complete. Further taxa screening is almost certain to reveal further details of this unfolding story.

A commonality among most GALV-KoRV-related retroviruses is their apparent shared use of the PiT-1 (SLC20A1) cellular receptor (O’Hara et al. 1990, Xu et al. 2013, 2015, Hayward et al. 2020). The use of PiT-1 for viral entry requires the presence of a permissive protein sequence motif (Schneiderman et al. 1996, Farrell et al. 2002, Hayward et al. 2020). The rodent *Mus musculus* does not possess this motif and is not susceptible to GALV-KoRV-related retroviruses (Hayward et al. 2020). It is reasonable to speculate that evolutionary pressure from PiT-1 receptor-utilizing retroviruses may lead to host adaptations to prevent viral entry, such as through mutations in receptor sequence motifs important for viral envelope interactions. We analysed the PiT-1 receptor sequences of the novel Australian and African rodent hosts of GALV-KoRV-related retroviruses through alignment against the PiT-1 receptor sequences of hosts known to be permissive or non-permissive to PiT-1-mediated retroviral infection (SI Figure 10). All of the novel rodent hosts except *Z. argurus* contained the permissive motif. *Z. argurus* possessed the same codon deletion in this motif that is present in the non-permissive motif of *M. musculus*; however, the other residues matched those of the permissive motif of *Rattus norvegicus*. It is unknown whether the codon deletion in *Z. argurus* is sufficient to prevent PiT-1-mediated viral entry, and this question should be investigated in future studies. Sustained evolutionary pressure that leads to host adaptations to prevent viral infection may suggest that rodents have contended with GALV-KoRV-related retroviruses for a significant evolutionary timeframe.

All rodent genera now known to have hosted GALV-KoRV-related retroviruses (*Melomys, Mastacomys, Pseudomys, Zyzomys, Mastomys, and Praomys*) are members of the Muridae family, within the order Rodentia, which diverged from other rodent families approximately at the start of the Miocene epoch ∼17–22 million years ago (Fabre et al. 2012, Aghová et al. 2018). The Australo-Papuan genera among these, *Melomys, Mastacomys, Pseudomys, Zyzomys*, are all members of the Hydromyini tribe (a taxonomic rank above genus, but below family and sub-family) which split from other Muridae ∼10.4 million years ago (Aghová et al. 2018). The GALV-KoRV-related retroviral sequences identified in the Australian rodent exomes are replete with deleterious frameshift and stop codon mutations, indicating that these ERVs integrated long ago. While it is not possible to accurately estimate the integration times of these short contigs without additional sequence data, other ERVs with similarly extensive mutations have been calculated to have integrated at times on the scale of millions of years ago (Martins et al. 2011, Hayward et al. 2013b).

Conversely, the African rodent genera, *Mastomys* and *Praomys*, members of the Praomyini tribe which radiated ∼6.8 million years ago (Aghová et al. 2018), contain ERVs with minimal or no obviously deleterious mutations and identical LTRs in the case of Mastomys WMV. In combination with the high percentage nucleotide identity with the Southeast Asian WMV, this suggests a much more recent transmission, and therefore integration time, than that for the Australo-Papuan rodents. If we consider human-mediated shipping as a means of transmission of retroviruses, it is reasonable to postulate that Mastomys WMV and Praomys WMV integrated as recently as within the last several hundred years.

These findings support the hypothesis that rodents are the primary host of GALV-KoRV-related retroviruses. While other mammals are clearly susceptible to infection with these gammaretroviruses, such transmission events may be incidental to the main thread of gammaretroviral divergence and evolution in rodent hosts. Bats are a potential exception. HPG appears to be endemic among Australian black flying foxes, and HPG’s closest relatives have been found within several different species of pteropid bats (Hayward et al. 2020, Van Brussel et al. 2023). Nucleic acid evidence reveals the presence of HPG or its very close relatives in a number of fruit bat species including *P. alecto, P. poliocephalus, Macroglossus minimus*, and *Syconycteris australis*. Serological evidence further indicates the presence of the HPG sub-clade in *P. conspicillatus*, and an Australo-Papuan microbat, *Rhinolophus megaphyllus* (Hayward et al. 2020, Van Brussel et al. 2023). The GALV-KoRV-related retroviruses of pteropid bats form a monophyly that does not contain any known rodent viruses, including the ones reported in this study (Hayward et al. 2020). This indicates that the HPG sub-clade has become well adapted to circulation among, and transmission between, different bat species. This successful adaptation has not yet been observed for GALV-KoRV-related retroviruses infecting other non-rodent clades of mammals.

### Hypothetical timeline of the GALV-KoRV-related retroviruses clade

A hypothetical timeline for the spread of GALV-KoRV-related retroviruses could feasibly involve ancestral origins within rodent hosts in the vicinity of mainland Asia or Southeast Asia, with divergence from other gammaretroviral lineages that currently include murine leukemia viruses (SI Figure 8A) hosted by Asian rodents. The GALV-KoRV-related retrovirus clade potentially emerged as a new lineage being transmitted through Southeast Asian/Australo-Papuan mammals, and eventually into Australian rodents. Transmission into rodent hosts on the Australian continent likely occurred on scale of millions of years ago, possibly across transient land bridges during periods of low ocean height and coincident with the radiation of the Hydromyini tribe of Australo-Papuan rodents (Aghová et al. 2018).

The existence of GALV-KoRV-related retroviruses on the Australian continent for millions of years may also help account for the both the large divergence of the KoRV sub-clade from the GALV/WMV sub-clade and the presence of HPG and its close relatives in Australian bats. Considering the possibility that the retroviral ancestors of extant KoRV and HPG diverged from GALV/WMV within Australia millions of years before endogenization in koalas, then in addition to transmission and adaptation to chiropteran and non-eutherian marsupial hosts, this may account for the relatively large sequence divergence between these retroviruses evident in phylogenetic analyses of the GALV-KoRV-related retrovirus clade (SI Figure 8A) (Denner 2016, Hayward et al. 2020, Mottaghinia et al. 2024).

Since arrival in Australia, this clade may have been transmitted back and forward between Australia and Southeast Asia by various natural and incidental hosts leading to the eventual integration of WMV variants in the genomes of Australo-Papuan rodents such as *Melomys* spp. and infections in the Southeast Asian microbats *Rhinolophus hipposideros* and *Hipposideros larvatus* (Hayward et al. 2020). Following arrival in Australia, one lineage that now includes HPG was transmitted into Australian fruit bats (Hayward et al. 2020, Van Brussel et al. 2023). The collective ranges of these species extend from Southeast Asia down through to South Australia.

**Figure 6.**
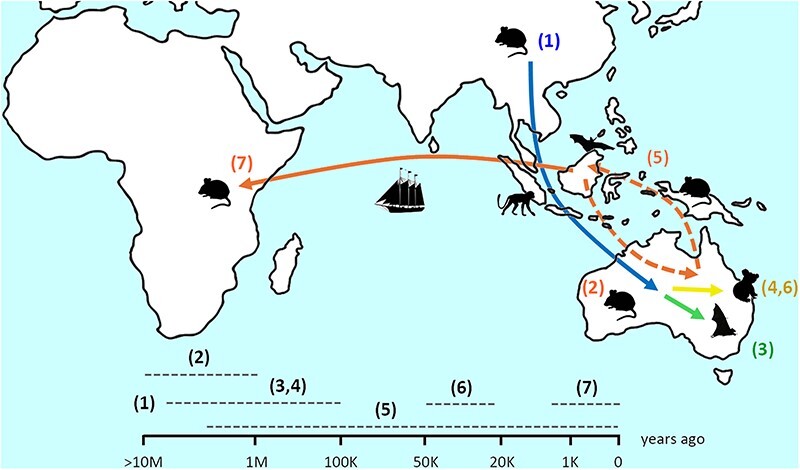
Hypothetical timeline of presently known key events in the evolutionary history of the GALV-KoRV-related retrovirus clade. (1) Potential ancestor(s) of the clade as gammaretroviruses hosted by Asian rodents on the scale of 10+ millions of years ago. (2) Emergence of the GALV-KoRV-related retrovirus clade as it is transmitted through Southeast Asian/Australo-Papuan mammals, and eventually into Australian rodents, on the scale of millions of years ago. (3,4) Diversification of the GALV-KoRV-related retrovirus clade by transmission into new hosts, leading to the emergence of modern lineages hosted by bats (3) and koalas (4), on the scale of hundreds of thousands to millions of years ago. (5) Bi-directional transmission of GALV-KoRV-related retroviruses through Australia, Papua New Guinea, and Southeast Asia, likely by natural rodent hosts with occasional transmission into incidental hosts including primates and bats, across the ongoing history of the clade. (6) The earliest potential beginning of endogenization of KoRV in the koala genome ∼22–50 thousand years ago. (7) Recent, potentially human-mediated transmission of a WMV-variant from the Australo-Papuan region into the African continent and new rodent hosts, on the scale of hundreds to >1000 years ago.

Similarly, the direct retroviral ancestor of KoRV was transmitted to koalas from another species, likely a rodent but possibly some other Australian mammal such as a bat. KoRV later began endogenization of the koala genome in the range of at most 22 200–49 900 years ago, with endogenization occurring multiple times since then until potentially quite recently (Ishida et al. 2015). Later, a WMV variant was transmitted to the African continent, possibly less than 1000 years ago, where it infected and endogenized in at least two rodent species. This hypothetical timeline is depicted in Fig. 6.

### Limitations of the study

An important limitation of this study is the lack of breadth of available sequence data. This is somewhat surprising considering that such large amounts of data already exist, but it is important to consider that for most species, data exist from a very limited number of individuals, and for many species no data exist at all. We might compare it to the analogy of trying to find a very specific type of human virus by analysing genomic and transcriptomic data from just a few people. The chances of success might be slim. It’s important to consider this when discussing the host range of GALV-KoRV-related retroviruses. While over recent years we have been discovering new host species (Simmons et al. 2014a, Alfano et al. 2016, McMichael et al. 2019, Hayward et al. 2020), and this study adds to that number, we may still have only scratched the surface. There may yet be any number of important animal species under threat from this group of cancer-causing viral pathogens.

The iterative search strategy employed in this study utilized a short 540 nt sequence representing the receptor binding domain of a GALV-KoRV-related retrovirus. It is important to note that some retroviruses are generated as the product of a recombination event between different, sometimes distantly related retroviruses (Negroni and Buc 2001, Hayward et al. 2013b). It is feasible that GALV-KoRV-related retroviruses exist which, as a result of recombination, possess a GALV-KoRV-related gag-pol region and env region from outside this clade or even this genus. This type of GALV-KoRV-related retrovirus would not be identified using this search strategy.

The contigs generated from Australian rodent exomes represent highly stringent, consensus short-read assemblies. It is possible that these rodents contain multiple GALV-KoRV-related ERVs that are highly similar. As such, it is possible that one or more contig assemblies could be generated from reads derived from multiple discrete ERVs. However, because these contigs would necessarily be derived from ERVs with a high percentage nucleotide identity, they still unambiguously demonstrate that these rodents have hosted/previously been infected by GALV-KoRV-related retroviruses. Future genome assemblies generated for these Australian rodents may help clarify this issue.

Regarding the potentially infectious nature of Mastomys WMV, it is important to emphasize that no modelling or infectivity experiments were performed, and as such infectivity has not been confirmed. While no obviously deleterious mutations such as frameshifting or stop codon mutations are present in Mastomys WMV, and all key sequence motifs are present and intact, other mutations such as non-synonymous mutations may have rendered this ERV non-infectious. Experimental approaches such as those conducted for HPG (Hayward et al. 2020) and cMWMV (Mottaghinia et al. 2024) should be undertaken to determine if Mastomys WMV is indeed infectious.

### Conclusion

The identification of numerous rodents as novel hosts of GALV-KoRV-related retroviruses widens our understanding of the host range of this clade of oncogenic viruses. It also highlights that our knowledge remains relatively limited. Many further animal species may already host these viruses or be susceptible to infection. The finding of GALV-KoRV-related retroviruses in African rodents, and the expanding association with rodent hosts more generally, demonstrates the transmissibility of these viruses and implicates rodents as an important host reservoir. Since GALV-KoRV-related retroviruses are associated with animal disease, future studies should seek to determine which animals could potentially become infected with them. Transmission of GALV-KoRV-related retroviruses from rodents or bats into animals of domestic, economic, or ecological importance could have dire consequences, as it has had for koalas.

## Methods

Our use of the term ‘GALV-KoRV-related retroviruses’ applies to retroviruses for which at least one of the three gammaretroviral genes *gag, pol*, or *env* are within this monophyly. This is because gammaretroviruses are known to undergo recombination (Bartosch et al. 2004, Anai et al. 2012, Hayward et al. 2013b, Henzy et al. 2014) and consequently may be directly derived from a GALV-KoRV-related retrovirus even if one or two of their genes are derived from other more distantly related retroviruses.

### Database searches

To identify previously unreported GALV-KoRV-related retroviruses within publicly accessible datasets, it was necessary to employ a strategy that would distinguish the target retroviral sequences from non-target retroviral sequences that would appear in our search results due to the presence of conserved sequence regions. Additionally, searches with large sequences, such as the complete KoRV genome, are computationally intensive and time consuming to run on public servers (Karasikov et al. 2024). Mammalian SRA records are too large and numerous to make comprehensive local analyses practical for all researchers. To account for these challenges, we used an iterative search strategy where our initial search query employed a short sequence from the least conserved region of the retroviral genome, the receptor binding domain within the *env* gene of a GALV-KoRV-related retrovirus. The rationale for this approach is that since there is a large degree of sequence diversity in the receptor binding domain, even among closely related retroviruses (Han et al. 1998, Xu et al. 2015, Hayward et al. 2020), any hit would likely be to a very close retroviral relative.

The search query was a 540-nt sequence from the receptor binding domain of HPG (SI Data 2). HPG is a GALV-KoRV-related retrovirus phylogenetically basal to KoRV and GALV (Hayward et al. 2020) (SI Figure 8A) and could be reasonably expected to possess homology to novel GALV-KoRV-related retroviruses also basal to the existing monophyly in addition to those phylogenetically intermediate to known GALV-KoRV-related retroviruses.

Searches were performed using the Sequence Read Archive Nucleotide BLAST (SRA BLAST) program through the NCBI web interface (https://blast.ncbi.nlm.nih.gov/Blast.cgi?PROGRAM=blastn&BLAST_PROGRAMS=megaBlast&PAGE_TYPE=BlastSearch&BLAST_SPEC=SRA&SHOW_DEFAULTS=on). Search parameters were left in their default settings except for the following: Optimized for ‘Somewhat similar sequences’; Word size = 7; Max target sequences = 1000; Max matches in a query range = 21; and Expect threshold = 1×10^−5^.

To identify GALV-KoRV-related endogenous retroviruses within genome assemblies, we searched the reference sequence (RefSeq) and whole genome shotgun (WGS) assemblies available in GenBank. Searches were performed for high-level taxa representing the breadth of the class Mammalia. We used the BLASTn program through the NCBI web interface (https://blast.ncbi.nlm.nih.gov/Blast.cgi) using the same search query as that for the SRA. Search parameters were left in their default settings except for the following: Database was set to ‘refseq_representative_genomes’, ‘refseq_genomes’, or ‘wgs’. Limited by organisms set to each of the ‘Search taxa’ listed in SI Table 3, Optimized for ‘Somewhat similar sequences’; Word size = 7; Max target sequences = 1000; and Expect threshold = 1×10^−20^.

### GALV-KoRV-related retrovirus contig assembly

To determine if the closest retroviral match for the identified SRA reads was a GALV-KoRV-related retrovirus, a local reciprocal BLAST analysis (Altschul et al. 1990) against known retroviruses was performed. SRA containing reads for which the closest match was a GALV-KoRV-related retrovirus were further analysed as follows: SRA BLAST searches used the genome region spanning the *gag, pol*, and *env* genes of the closest matching retrovirus as the search query. Search parameters were left in their default settings except for the following: Optimized for ‘Somewhat similar sequences’; Word size = 7; Max target sequences = 1000; Max matches in a query range = 21; and Expect threshold = 1×10^−5^.

Matching SRA reads were collected and underwent *de novo* assembly into contigs using the Assemble Sequences tool in CLC Genomics 22 (CLC; QIAGEN) with default parameters except Alignment Stringency = High and Minimum aligned read length = 10 (SI Data 1). A local BLAST analysis against known retroviruses was performed for the assembled contigs to identify those whose closest match was a GALV-KoRV-related retrovirus.

### Annotation of mastomys WMV genome

The Mastomys WMV genome sequence was annotated using CLC following alignment using MUSCLE (Edgar 2004), and comparison against the genomes of WMV (Genbank: KT724051) and HPG (Genbank: MN413610).

### Phylogenetic analyses

To estimate the evolutionary relationships between the endogenous retroviral sequences identified in genome assemblies and SRA with known gammaretrovirus, we conducted phylogenetic analyses. Endogenous retroviruses were extracted from genome assembly contigs by delineation of their long-terminal repeats as described previously (Hayward et al. 2013b). The *gag, pol*, and *env* genes were identified and annotated by sequence alignment against known gammaretrovirus genes using MUSCLE followed by manual curation. Contigs/reads from SRA were aligned to the *gag, pol*, and *env* genes of known gammaretroviruses using the progressive alignment tool in CLC with the following parameters: Gap open cost = 5; Gap extension cost = 2; End gap cost = free; Alignment speed = ‘Very accurate’. Contigs derived from *Pseudomys delicatulus* (*env*) and *P. shortridgei* (*env*) did not overlap any other contigs or reads; in these cases, adjacent contigs were concatenated to improve phylogenetic resolution. Ambiguous regions of the alignments were removed using Gblocks (Castresana 2000).

The best-fit models of nucleotide substitution were determined using the Model Testing tool in CLC, which were found to be GTR + G + T for all alignments. Maximum likelihood trees were then inferred using the best-fit model in CLC with 1000 bootstrap replicates. Trees were visualized with MEGA 11.0 (Tamura et al. 2021).

### PiT-1 sequence analysis

PiT-1 (SLC20A1) genes for species with assembled genomes were extracted from GenBank (SI Table 3). For each of the rodents without assembled genomes, the PiT-1 gene was assembled from the same SRA data set that the GALV-KoRV-related retroviral sequences were extracted as follows: The PiT-1 gene of the rodent, *Mastomys coucha* (GenBank: XM_031371865), was used as a search query. An SRA BLAST was conducted with search parameters left in their default settings except for the following: Optimized for ‘Highly similar sequences’; Word size = 16; Max target sequences = 1000; and Expect threshold = 1×10^−20^. Reads were *de novo* assembled in CLC and the resultant contig confirmed to encode PiT-1 by reciprocal BLAST analysis and sequence alignment using MUSCLE.

## Supplementary Material

veae061_Supp

## Data Availability

The novel GALV-KoRV-related retroviral contigs reported in this study are provided as SI Data 1. GenBank accessions for the publicly available sequence data used in this study are listed in Tables 1 and 2, and SI Table 4. Mastomys WMV and Praomys WMV nucleotide sequences have been deposited in GenBank, with accessions PP852712 and PP852713.
