# Supplementary material for: GALV-KoRV-related retroviruses in diverse Australian and African rodent species
Source: Virus Evol. 2024 Jul 31;10(1):veae061. doi: 10.1093/ve/veae061 (PMC11341202; doi:10.1093/ve/veae061)
Supplement: veae061_Supp [file veae061_supp.zip › suppl_data/SI Figures.docx]

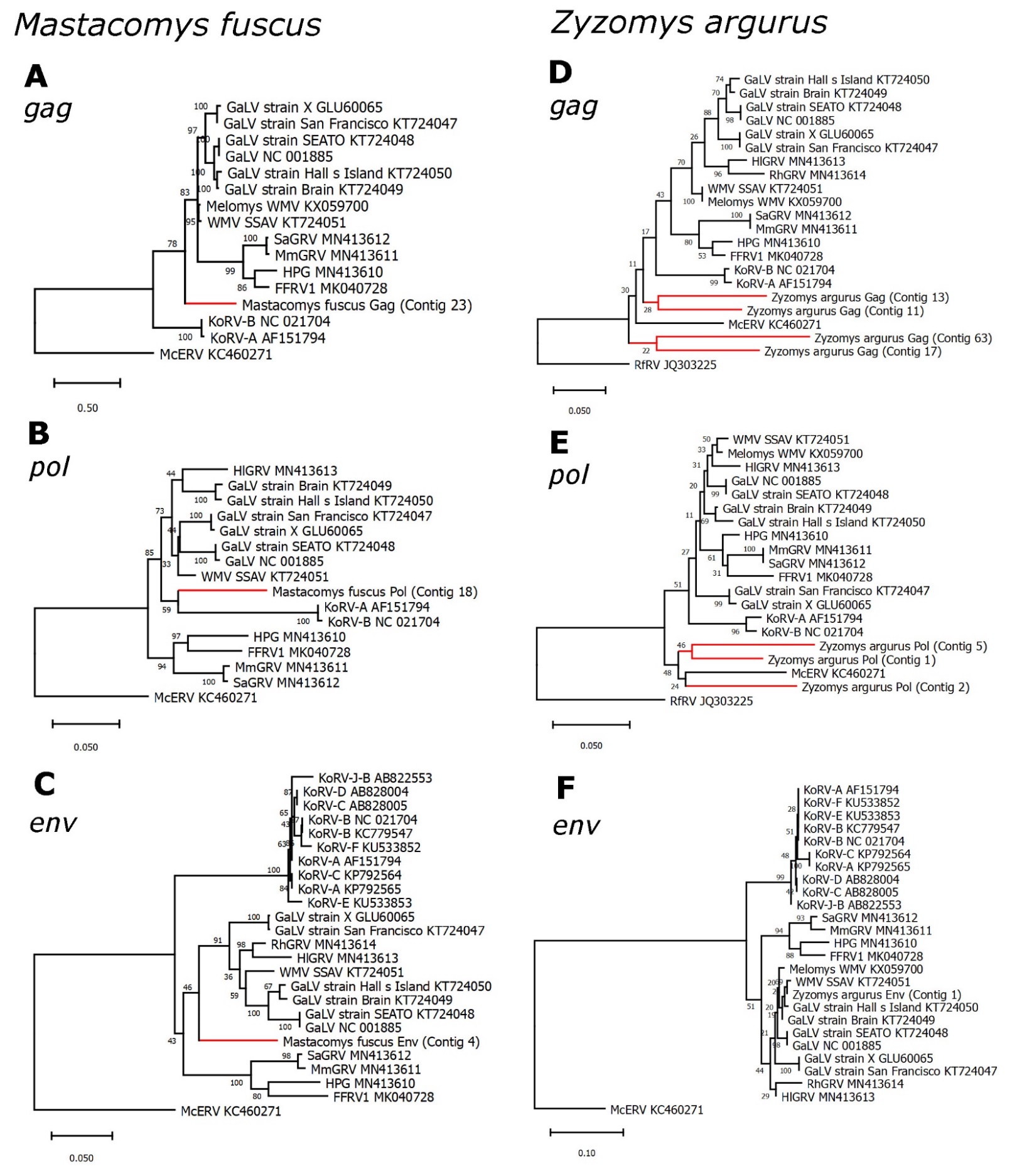


**SI Figure 1. Phylogenetic evolutionary analysis of Australian *Mastacomys fuscus* and *Zyzomys argurus* GALV-KoRV-related *gag pol* and *env* retroviral sequences.** Maximum likelihood phylogenies of regions of nucleotide sequences from the **A, D** *gag*, **B, E** *pol*, and **C, F** *env* genes overlapping contigs from **A-C** *M. fuscus* and **D-F** *Z. argurus*. All branches are scaled according to the number of nucleotide substitutions per site as indicated by the scale bars. Trees were rooted using the McERV (*Mus caroli* endogenous retrovirus) KC460271 or RfRV (*Rhinolophus ferrumequinum* retrovirus) JQ303225 sequences. Bootstrap support values are shown at the nodes. The number of nucleotide positions in the multiple sequence alignments used to generate phylogenies A-F are 776, 794, 523, 154, 194, and 175, respectively.


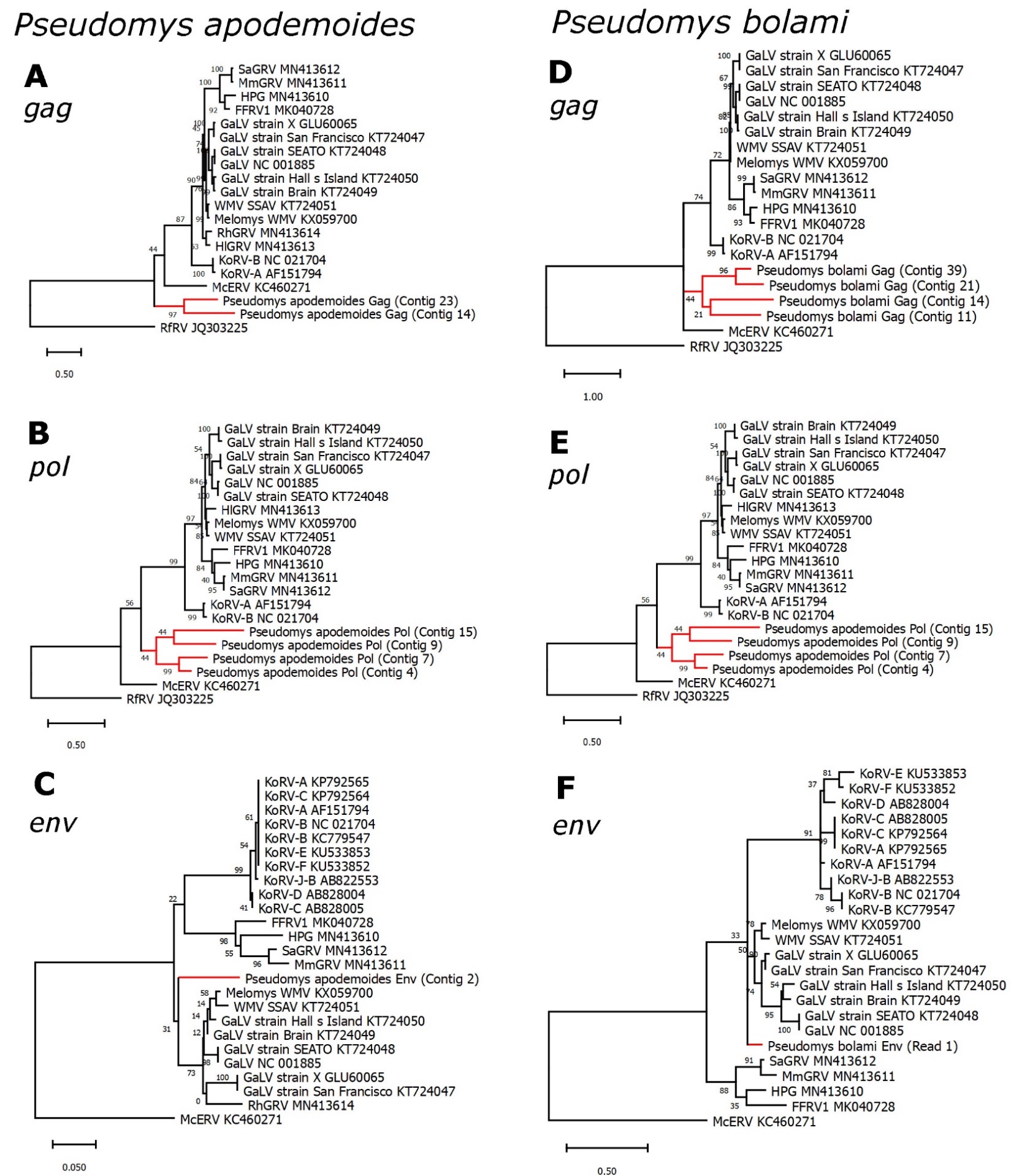


**SI Figure 2. Phylogenetic evolutionary analysis of Australian *Pseudomys apodemoides* and *P. bolami* GALV-KoRV-related *gag pol* and *env* retroviral sequences.** Maximum likelihood phylogenies of regions of nucleotide sequence from the **A, D** *gag*, **B, E** *pol*, and **C, F** *env* genes overlapping contigs from **A-C** *P. apodemoides* and **D-F** *P. bolami*. All branches are scaled according to the number of nucleotide substitutions per site as indicated by the scale bars. Trees were rooted using the McERV (*Mus caroli* endogenous retrovirus) KC460271 or RfRV (*Rhinolophus ferrumequinum* retrovirus) JQ303225 sequences. Bootstrap support values are shown at the nodes. The number of nucleotide positions in the multiple sequence alignments used to generate phylogenies A-F are 663, 413, 172, 206, 183, and 198, respectively.


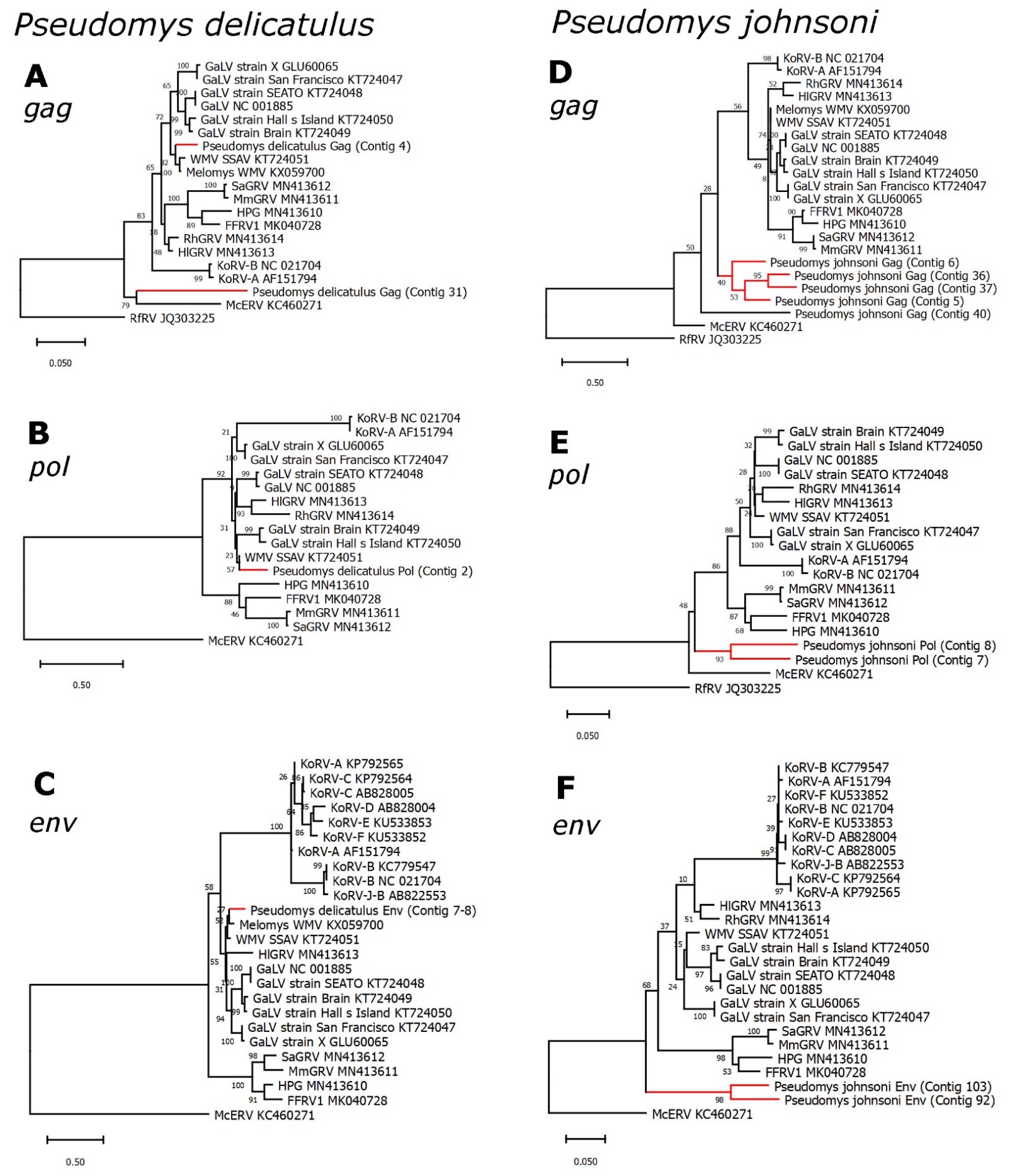


**SI Figure 3. Phylogenetic evolutionary analysis of Australian *Pseudomys delicatulus* and *P. johnsoni* GALV-KoRV-related *gag pol* and *env* retroviral sequences.** Maximum likelihood phylogenies of regions of nucleotide sequence from the **A, D** *gag*, **B, E** *pol*, and **C, F** *env* genes overlapping contigs from **A-C** *P. delicatulus* and **D-F** *P. johnsoni*. All branches are scaled according to the number of nucleotide substitutions per site as indicated by the scale bars. Trees were rooted using the McERV (*Mus caroli* endogenous retrovirus) KC460271 or RfRV (*Rhinolophus ferrumequinum* retrovirus) JQ303225 sequences. Bootstrap support values are shown at the nodes. The number of nucleotide positions in the multiple sequence alignments used to generate phylogenies A-F are 589, 469, 476, 444, 306, and 150, respectively.


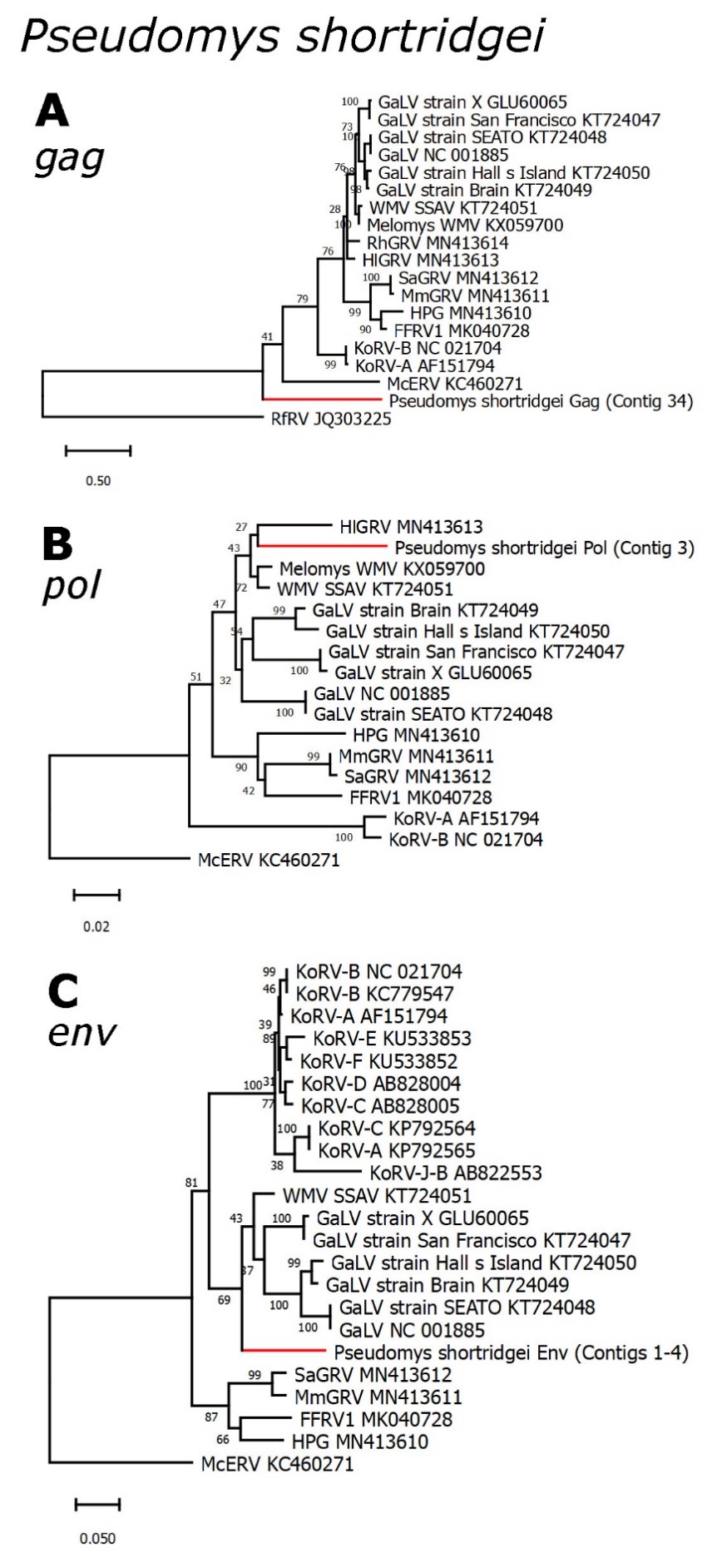


**SI Figure 4. Phylogenetic evolutionary analysis of Australian *Pseudomys shortridgei* *gag pol* and *env GALV-KoRV-related* retroviral sequences.** Maximum likelihood phylogenies of regions of nucleotide sequence from the **A** *gag*, **B** *pol*, and **C** *env* genes overlapping contigs from *P. shortridgei*. All branches are scaled according to the number of nucleotide substitutions per site as indicated by the scale bars. Trees were rooted using the McERV (*Mus caroli* endogenous retrovirus) KC460271 or RfRV (*Rhinolophus ferrumequinum* retrovirus) JQ303225 sequences. Bootstrap support values are shown at the nodes. The number of nucleotide positions in the multiple sequence alignments used to generate phylogenies A-C are 567, 279, and 445, respectively.


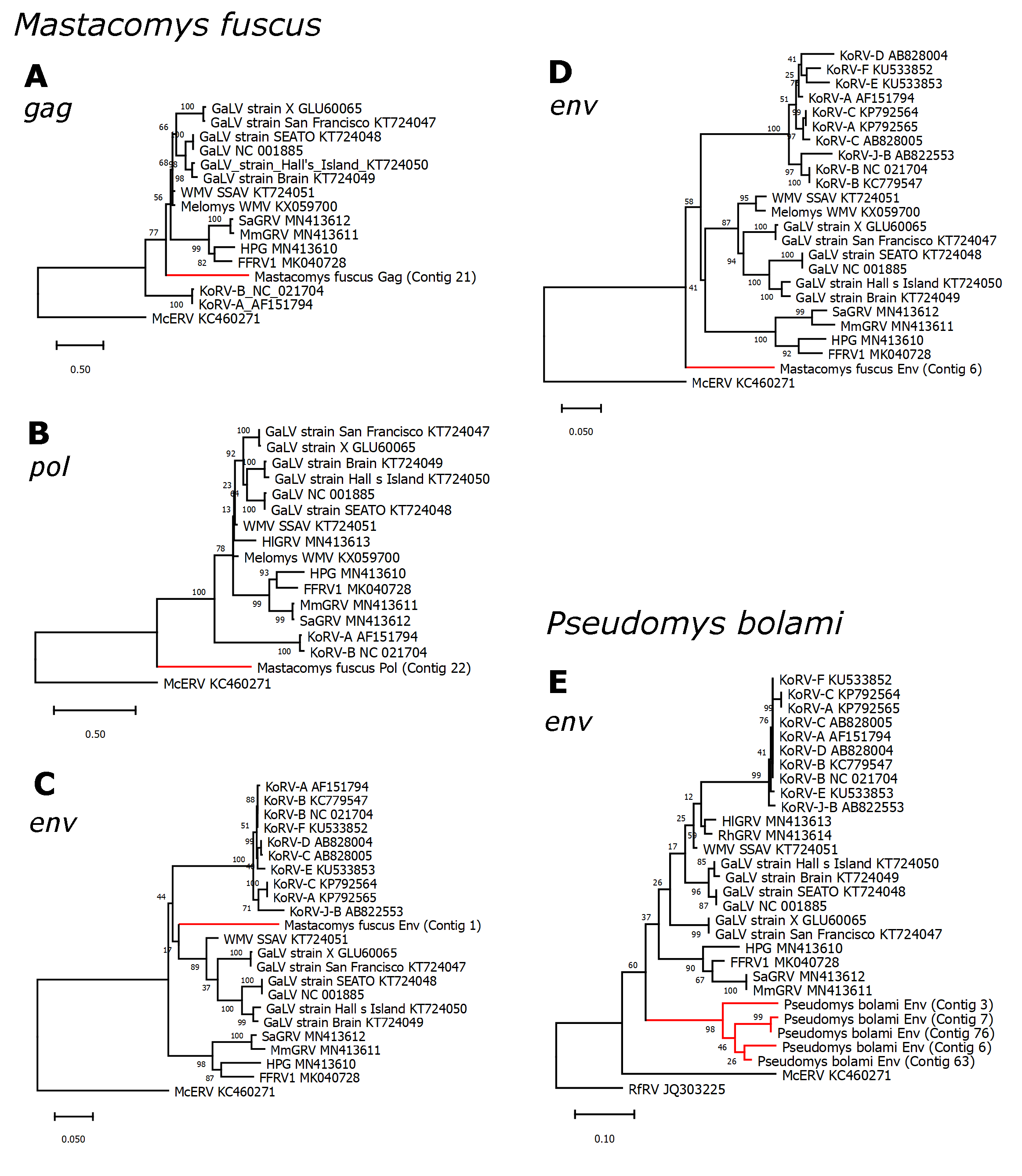


**SI Figure 5. Phylogenetic evolutionary analysis of additional *Mastacomys fuscus* and *Pseudomys bolami* GALV-KoRV-related retroviral sequences.** Maximum likelihood phylogenies of regions of nucleotide sequence from the **A** *gag*, **B** *pol*, and **C, D, E** *env* genes overlapping contigs from **A-D** *M. fuscus* and **E** *P. bolami*. All branches are scaled according to the number of nucleotide substitutions per site as indicated by the scale bars. Trees were rooted using the McERV (*Mus caroli* endogenous retrovirus) KC460271 or RfRV (*Rhinolophus ferrumequinum* retrovirus) JQ303225 sequences. Bootstrap support values are shown at the nodes. The number of nucleotide positions in the multiple sequence alignments used to generate phylogenies A-E are 397, 840, 647, 450, and 108, respectively.


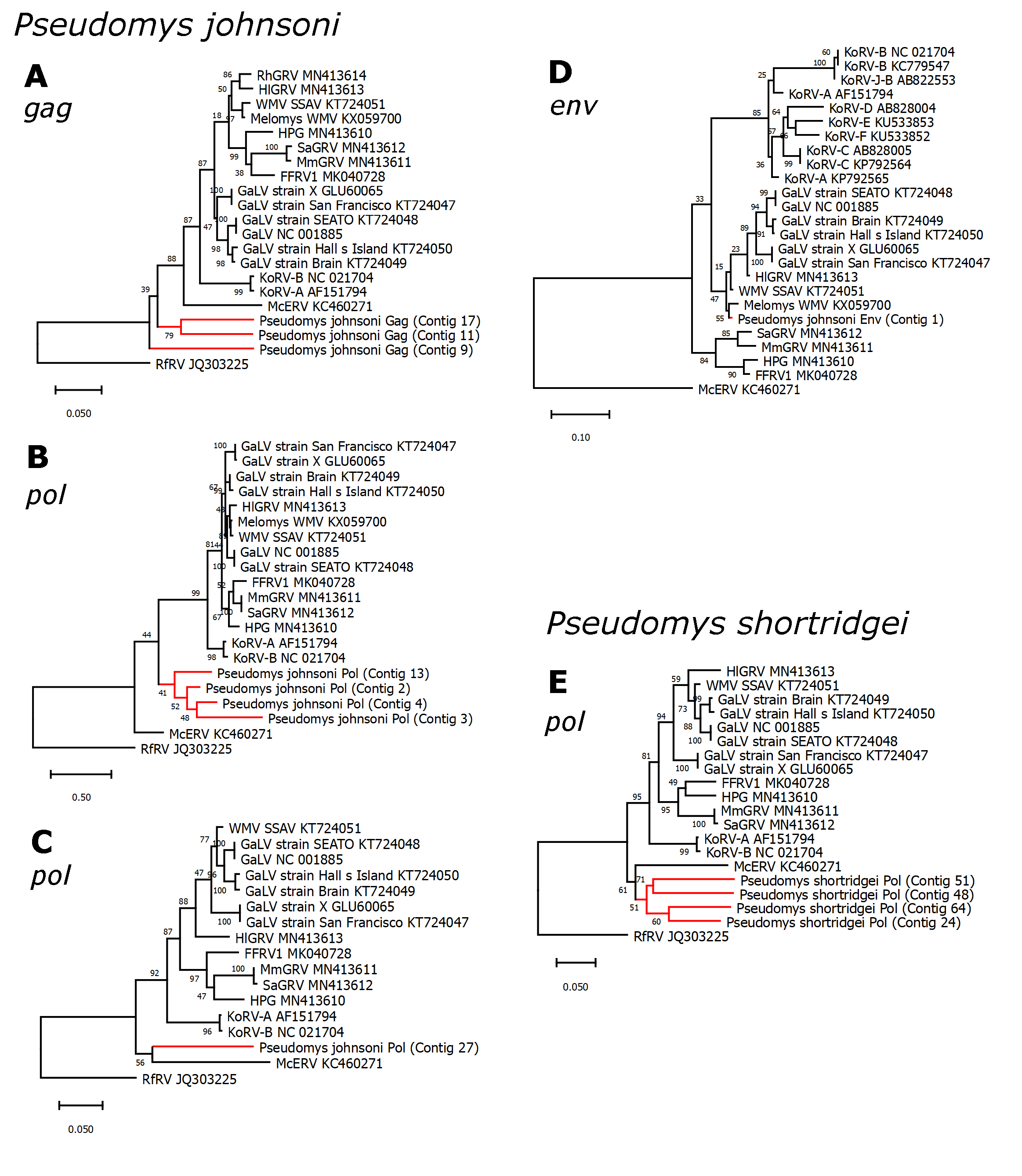


**SI Figure 6. Phylogenetic evolutionary analysis of additional *Pseudomys johnsoni* and *P. shortridgei* GALV-KoRV-related retroviral sequences.** Maximum likelihood phylogenies of regions of nucleotide sequence from the **A** *gag*, **B, C, E** *pol*, and **D** *env* genes overlapping contigs from **A-D** *P. johnsoni* and **E** *P. shortridgei*. All branches are scaled according to the number of nucleotide substitutions per site as indicated by the scale bars. Trees were rooted using the McERV (*Mus caroli* endogenous retrovirus) KC460271 or RfRV (*Rhinolophus ferrumequinum* retrovirus) JQ303225 sequences. Bootstrap support values are shown at the nodes. The number of nucleotide positions in the multiple sequence alignments used to generate phylogenies A-E are 210, 284, 476, 146, and 403, respectively.


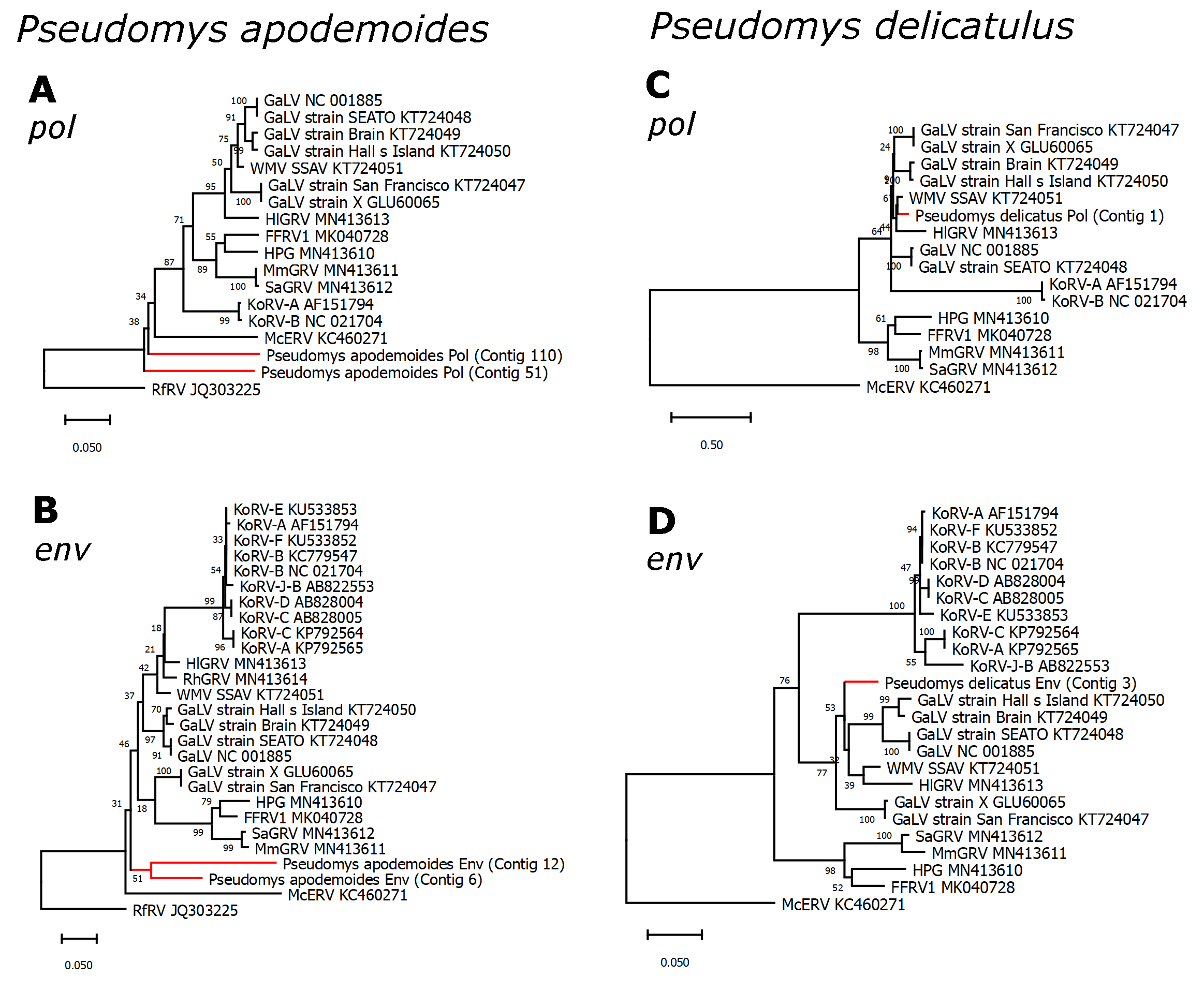


**SI Figure 7. Phylogenetic evolutionary analysis of additional *Pseudomys apodemoides* and *P. delicatulus* GALV-KoRV-related retroviral sequences.** Maximum likelihood phylogenies of regions of nucleotide sequence from the **A, C** *pol*, and **B, D** *env* genes overlapping contigs from **A, B** *P. apodemoides* and **C, D** *P. delicatulus*. All branches are scaled according to the number of nucleotide substitutions per site as indicated by the scale bars. Trees were rooted using the McERV (*Mus caroli* endogenous retrovirus) KC460271 or RfRV (*Rhinolophus ferrumequinum* retrovirus) JQ303225 sequences. Bootstrap support values are shown at the nodes. The number of nucleotide positions in the multiple sequence alignments used to generate phylogenies A-D are 426, 160, 950, and 377, respectively.


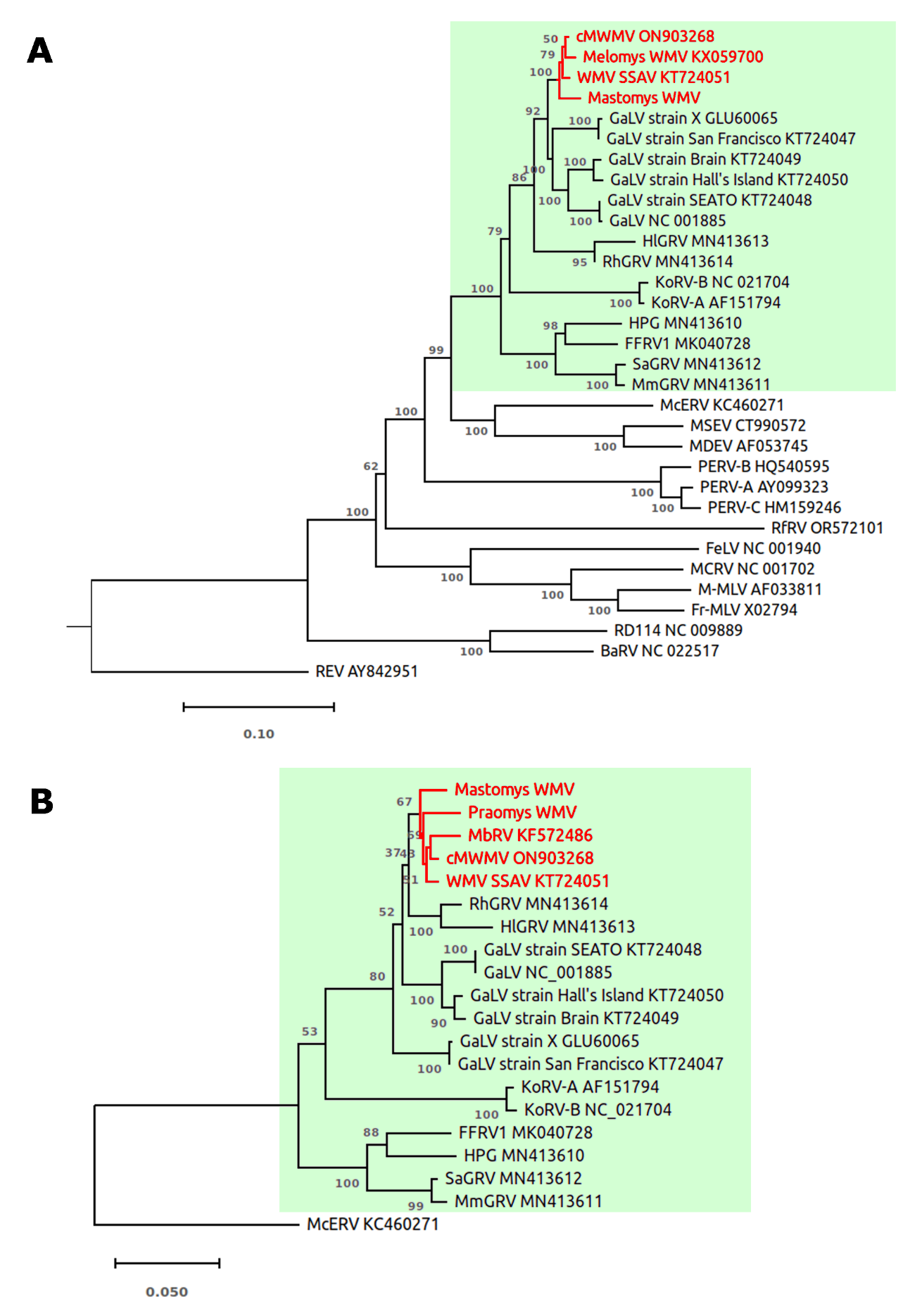


**SI Figure 8. Maximum likelihood phylogenetic evolutionary analysis of (A) gammaretroviral genome nucleotide sequences and (B) a partial sequence phylogeny including *Melomys burtoni* retrovirus (MbRV)*.*** The GALV-KoRV-related retrovirus clade is shaded in green, and the WMV sub-clade is highlighted in red text. All branches are scaled according to the number of nucleotide substitutions per site as indicated by the scale bars. Trees were rooted using the REV (Reticuloendotheliosis virus) AY842951 or McERV (*Mus caroli* endogenous retrovirus) KC460271 sequences. Bootstrap support values are shown at the nodes. The number of nucleotide positions in the multiple sequence alignments used to generate phylogenies A-B are 6430 and 601, respectively.


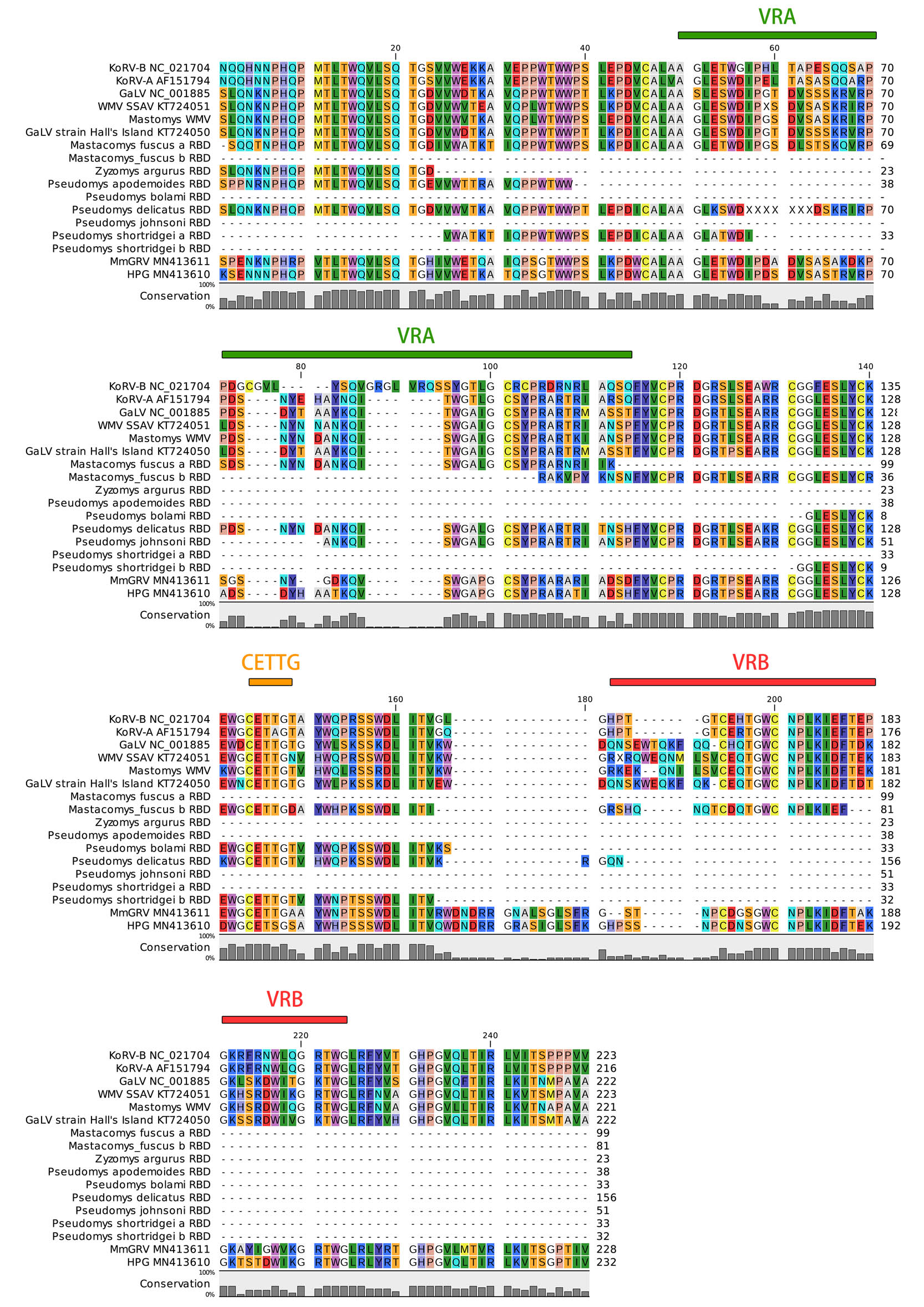


**SI Figure 9. Multiple sequence alignment of the receptor binding domain of novel rodent GALV-KoRV-related retroviruses.** The alignment was generated using MUSCLE (1). Novel sequences are compared against several known sequences. Highly variable regions A (VRA) and B (VRB) indicated by the green and red lines, respectively. The CETTG motif is denoted with an orange line. Due to the limited nature of the available sequences, not all sequences span the entire region.


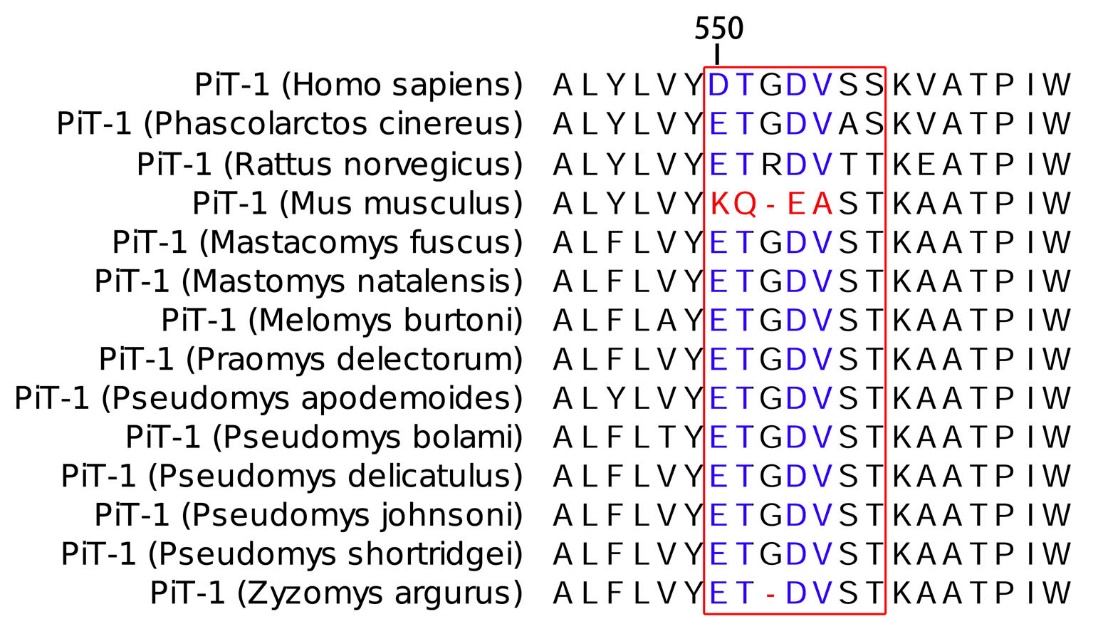


**SI Figure 10. Multiple sequence alignment of residues of the PiT-1 Region A of novel rodent hosts of GALV-KoRV-related retroviruses.** The Region A ‘permissivity’ motif of mammalian PiT-1 (SLC20A1) is shown in the red box (amino acid positions 550-557). Residues highlighted in blue and red denote residues in infection susceptible and resistant mammalian PiT-1 homologs, respectively (2).

## References

1. Edgar RC. 2004. MUSCLE: multiple sequence alignment with high accuracy and high throughput. Nuc Acid Res 32:1792-1797.

2. Farrell KB, Russ JL, Murthy RK, Eiden MV. 2002. Reassessing the role of region A in Pit1-mediated viral entry. J Virol 76:7683-7693.
